# Establishment of feijoa (*Acca sellowiana*) callus and cell suspension cultures and identification of arctigenin - a high value bioactive compound

**DOI:** 10.3389/fpls.2023.1281733

**Published:** 2024-01-17

**Authors:** Sanjeev V. Raikar, Ilena Isak, Sunita Patel, Harriet L. Newson, Stefan J. Hill

**Affiliations:** New Zealand Forest Research Institute Ltd. (Scion), Rotorua, New Zealand

**Keywords:** feijoa, callus, cell suspensions, elicitors, bioactive compounds

## Abstract

Feijoa (*Acca sellowiana (O. Berg.) Burret*), also known as pineapple guava, is a member of the Myrtaceae family and is well known for its fruit. Chemical profiling of the different tissues of the feijoa plant has shown that they generate an array of useful bioactive compounds which have health benefits such as significant antioxidant activities. In this study, an *in vitro* culture system has been developed, which could be explored to extract high-value bioactive compounds from feijoa. Feijoa tissue culture was initiated by the induction of callus from floral buds. Sections of floral buds were plated on MS medium supplemented with 2,4-D and BAP at 2.0mg/L and 0.2mg/L concentrations, respectively. Cell suspension cultures of feijoa were established using a liquid MS medium with different concentrations of 2,4-D and BAP and cultured on a rotary shaker. The growth of the cell suspension was evaluated with different parameters such as different carbohydrate sources, concentration of MS media, and inoculum density. When the cell suspensions were treated with different concentrations of MeJA at different time points, phytochemicals UPLC - QTOF MS analysis identified extractables of interest. The main compounds identified were secondary metabolites (flavonoids and flavonoid-glucosides) and plant hormones. These compounds are of interest for their potential use in therapeutics or skin and personal care products. This report investigates essential methodology parameters for establishing cell suspension cultures from feijoa floral buds, which could be used to generate *in vitro* biomass to produce high-value bioactive compounds. This is the first study reporting the identification of arctigenin from feijoa, a high-value compound whose pharmaceutical properties, including anti-tumour, anti-inflammatory and anti-colitis effects, have been widely reported. The ability of feijoa cell cultures to produce such high-value bioactive compounds is extremely promising for its use in pharmaceuticals, cosmeceuticals and nutraceuticals applications.

## 1 Introduction

Feijoa (*Acca sellowiana* (O. Berg) Burret), also known as pineapple guava, is a member of the Myrtaceae family and is native to South America. Feijoa is widely grown in regions like New Zealand, Australia and some parts of the USA for its highly aromatic fruit. Recently, research into the bioactivity of feijoa fruit has significantly appreciated its pharmacological value. Patents have been published on the commercial application of extracts of feijoa for dietary supplements, which alleviate inflammation and pain (Nair et al., 2004) as well as the use of feijoa extract as a lipid antioxidant (Ishigami et al., 1998) and suppress the absorption of cholesterol (Onodera et al., 1998). These research works indicate the production of useful bioactive compounds from different parts of the feijoa plant. New triterpene acids were described by Verardo et al. (Verardo et al., 2019) while analysing callus developed from the fruit pulp of feijoa could have potential applications in human health. Mokhtari et al. (2018) identified high-value antifungal compounds from the peel of feijoa fruit, which inhibit chitin synthesis after screening several feijoa varieties and have been effective against *Candida* species which cause most fungal infections in humans. Montoro et al. (2020) also identified bioactive compounds from feijoa flowers that can be used to prepare nutraceuticals and dietary supplements. Recently, Amer et al. (2023) identified antithrombotic, anti-oxidant and anti-inflammatory properties from feijoa extracts isolated from callus and cell biomass from cell cultures in a bioreactor using biological assessments through *in-vivo* studies. The fruit extract has also shown bactericidal activity against gram-negative bacteria such as *Pseudomonas*, *Enterobacter* and *Salmonella* (Vuotto et al., 2000). There have been reports about a significantly higher concentration of abscisic acid than other fruit plants, which has been identified as a novel nutraceutical for controlling glycaemic index (Zocchi et al., 2017). These properties of feijoa are already being exploited by commercial entities for marketing feijoa fruit extract as a potential immune-boosting agent against diseases such as type II diabetes.

However, the practicality of using the identified bioactive compounds towards their intended use is restricted since these compounds are found in very low concentrations, and their production depends on many biotic and abiotic factors. Furthermore, these compounds tend to be complex, which could require highly complex extraction and purification processes. Recently, a lot of focus has been on developing alternative methods of generating biomass for extraction of high-value compounds, such as the application of large bioreactors (to the scale of 1000L and more) and utilisation of the biomass for various purposes such as extraction of high-value compounds, as food substitutes with the aim of reducing environmental footprint and maintaining the ecological balance (Nordlund et al., 2018; Rischer et al., 2020). Private investment in this technology has resulted in the successful production of drugs such as Paclitaxel and Docetaxel by Phyton Biotech, and Taliglucerase Alfa by Protalix Biotherapeutics (Häkkinen et al., 2020). Plant cell and organ cultures have been considered as a potential source to produce biomass for the synthesis of bioactive compounds, which can be used in various applications, including pharmaceuticals, nutraceuticals and cosmeceuticals production and tissue culture techniques such as cell cultures in bioreactors. Plant tissue culture is a perpetual source for producing industrially important bioactive compounds (Chandran et al., 2020). The production of ginsenoside from *Panax ginseng* (Sivakumar et al., 2005), stevioside from *Stevia rebaudiana* cell suspension culture (Mathur and Shekhawat, 2013), ajmalicine from *Catharanthus roseus* (Lee and Shuler, 2000) shikonin from *Lithospermum erythrorhizon*, berberine from *Coptis japonica* (Fujita, 2007) rosmarinic acid from *Orthosiphon stamineus* and *Coleus blumei* (Ulbrich et al., 1985; Liang et al., 2006) and sanguinarine from *Papaver somniferum* (Eilert et al., 1985) are some examples where different types of plant tissue culture systems have been used to generate biomass for the extraction of bioactive compounds. These techniques provide options to circumvent the limitations for producing useful bioactive compounds (Robles-Martínez et al., 2016; Krasteva et al., 2021). Advances in plant genomics and metabolomics have also made it possible to better understand bioactive compounds’ dynamics in terms of their properties controlling factors which could result in improved production systems. This paper focuses on the effect of different parameters investigated in the generation of *in vitro* cell suspension culture system for feijoa floral buds, which could be used to generate cell biomass to synthesise secondary metabolites. This study also describes identifying bioactive compounds of significant importance from feijoa tissue and cell cultures, which could be explored for their potential application in various fields.

In this paper we demonstrate a possible route to explore the production of bioactive compounds from feijoa through the generation of cell biomass using an *in vitro* cell suspension culture method.

## 2 Materials and methods

### 2.1 Plant material

Feijoa plant material was collected from a Rotorua, New Zealand home garden. Floral buds were collected in December 2021. The floral buds were clipped from the tree and placed on ice in a polystyrene box. Sterilisation of the buds was done by initially treating the buds with double distilled water. The buds were then briefly immersed in 70% ethanol for 30 seconds and were immediately transferred to calcium hypochlorite 2% with 0.5 µL of Tween80 for 3 min. The material was finally washed with double distilled water (3 times) before plating on Murashige Skoog (MS) medium (Murashige and Skoog, 1962).

### 2.2 Callus induction and cell suspension initiation

The floral buds were segmented into 3 regions, viz. the top, the middle and the bottom region and cultured on MS medium in Petri plates (100 mm diameter; 20 mm depth). MS medium, supplemented with 30g/L sucrose and 3g/L agar with different concentrations of plant growth regulators (PGR) viz., 2,4-D at 0.2, 2.0, and 5.0 mg/L, and BAP at 0.2 mg/L was used to evaluate the response of the tissue material for callus induction. For each treatment, 5 plates of each floral region with 6 explants per plate were set up to evaluate callus induction. Callus induction was assessed as the fresh weight of the callus induced at each combination of PGR concentrations.

To establish the cell suspension cultures, 8-10 weeks old friable callus maintained by subculturing every 2 weeks was selected. One gram fresh weight (FW) of friable callus was transferred to 30 ml of MS medium supplemented with 2 mg/L 2,4-dichlorophenoxy acetic acid (2,4-D) and 0.2 mg/L benzylamino purine (BAP) in 150 ml Erlenmeyer flask without agar. The flasks were placed on an orbital shaker at 105 rpm and cultured in the dark at 22°C. The subculture cycle was maintained at 12 days. The cell suspensions were optimised by experimenting with the strength of MS media, the concentration of sucrose in the medium, the inoculum density, supplementing MS media with different sugars and varying the 2,4-D concentration.

### 2.3 Growth curve experiment

The cells (1 g FW) were cultured in 150 mL Erlenmeyer flasks containing 30 ml of MS medium supplemented with 2 mg/L 2,4-D and 0.2 mg/L BAP. Cultures were placed on a rotary shaker set at 105 rpm in a culture room maintained at 22°C in the dark. Cells from each flask were harvested by suction-filtering through a filter paper (Whatman filter paper No. 15cm diameter) layered in a Büchner funnel connected to a vacuum. The cells were harvested every 5 days for a duration of 35 days, and the fresh weight was determined. Cells were harvested from three flasks for each measurement. The optimum day for subsequent subcultures was determined based on the growth curve.

### 2.4 Cell suspension optimisation by varying strength of MS medium, sucrose and 2,4-D concentrations, inoculum density and different carbon sources

The cell suspension was cultured at different strengths of MS medium (half, three-fourths and full strength). The effect of sucrose concentration in MS medium on cell suspension growth was evaluated at 3 different concentrations viz. 2%, 3% and 4%. Cell suspension growth was also measured at 3 different concentrations of 2,4-D (1, 2, 4 mg/L) and in MS medium without any PGR. Different carbon sources at 3% concentration (sucrose, glucose, fructose and maltose) were evaluated for their effect on cell suspension growth using full-strength MS medium supplemented with 2,4-D and BAP at 2 mg/L and 0.2 mg/L, respectively. All the above experiments were initiated with an inoculum density of 3 g FW/100 ml and were cultured over a period of 30 days. Three different inoculum densities of the cell suspension (1.5, 3, and 6 g FW/100 ml) were investigated for their effect on the growth of the cell suspension in full-strength MS medium supplemented with sucrose 3% and 2,4-D and BAP at 2 mg/L and 0.2 mg/L respectively.

The growth of the cell suspensions in all the above experiments was measured after 30 days and was evaluated by calculating the percentage growth, which was calculated as:


$$\text{Percentage Growth}=\frac{Final\;fresh\;weight-Initial\;fresh\;weight}{Initial\;fresh\;weight}\times 100$$

To determine the fresh weight of the cells, the cell suspension was vacuum filtered with Whatman filter paper in a Büchner funnel connected to a vacuum and the weight of the cells collected was measured.

### 2.5 Elicitation in cell suspensions with methyl jasmonate

The cell suspensions were treated with 2 different concentrations of MeJA (50 and 100 µM) during the elicitation experiments. Fresh cell suspensions were initiated in 250 mL Erlenmeyer flasks with 2 g FW of cells transferred to 75 mL of MS medium supplemented with sucrose at 3%, 2,4-D and BAP at 2 and 0.2 mg/L, respectively. The cultures were placed on an orbital shaker at 105 rpm at 22°C in the dark. The cell suspension was treated with 50 and 100 µM MeJA on 3 different days, viz. 20, 25 and 30 days after initiation. The cell suspension samples (1.5 mL) treated with MeJA were collected in Eppendorf tubes for phytochemical analysis at different times (0, 6, 12, 24, 48 and 72 hours after treatment with MeJA) each day. The samples were stored in a -80°C freezer until the analysis.

### 2.6 Phytochemical liquid chromatography mass spectrometry analysis

Cell suspension samples were thawed at room temperature and centrifuged for 5 min at 3000 rpm. The cell pellets (approximately 50 mg) were used for extraction, and the supernatant was stored separately for further investigations. The extraction was done by re-suspending cell pellets in 1 mL 80:20 methanol/water (Merck HPLC grade) with vortex mixing; extraction solvent was used as blank. The samples were sonicated for 45 min then centrifuged at 3900 rpm for 10 mins. 1 mL of extracts and blanks were filtered with a PTFE syringe filter, transferred into a glass vial, and analysed directly. Floral buds frozen samples were thawed at room temperature, and 600 µl of 50:50 methanol/water was added in order to extract polar compounds. Samples were incubated into a shaker at 37°C at 150 rpm for 15 mins, centrifuged at 13000 rpm for 2 mins, then 300 µl of chloroform were added and incubated at 37°C at 1500 rpm for 10 mins and centrifuged at 13000 rpm for 5 mins. The polar fraction of the supernatant was transferred into a glass vial, and extracts were dried using a Genevac EZ-2 plus evaporating system. Samples were re-suspended in 500 µl methanol and analysed directly. Arctigenin commercial standard was purchased from Cayman Chemical Company, was dissolved in methanol at a concentration of 100 ppb (parts per billion) and directly analysed as per the method below.

Samples of cell suspension, floral buds and arctigenin standard were analysed on an Agilent ultra-high performance liquid chromatography quadrupole time of flight mass spectrometer (UPLC – QTOF MS) equipped with an Agilent 1290 high-speed binary pump, Agilent 1290 multisampler, Agilent 1290 multi-column thermostat, Agilent 1260 diode array detector, Dual AJS ESI source and 6545XT QTOF System, using the following conditions: Phenomenex Kinetex Evo C18 150 x 3.0 mm 2.6 um column; solvent gradient was Solvent A (water + 0.1% formic acid) and solvent B (acetonitrile + 0.1% formic acid), flow rate was 0.5 mL/min and temperature was kept at 30°C. The run time was 16 minutes with 6 minutes post-time. The acquisition was performed in negative polarity, and the sample injection was 1 µL. UPLC gradient details and the mass spectrometer detector parameter are shown in **Supplementary Tables S1** and **S2**.

### 2.7 UPLC – QTOF MS data processing

The data was processed using Agilent MassHunter Qualitative analysis software. The UPLC – QTOF MS data was screened against a copy of the PlantCyc database (curated by the Plant Metabolomics Network (PMN), www.plantcyc.org). The Target/Suspect Screening, Find by Formula function, was used with an absolute peak height filter of 20,000 counts and a quality score above 70.00. The mass tolerance was set at +/- 5 ppm. The hits were manually screened, and the best identification was selected based on the highest score, best extracted ion chromatogram (EIC) peak shape, and likely presence in the sample. The details of the compounds identified – including mass, m/z and EIC peak area were exported to Excel and then sorted by average peak area over the replicates to identify the putatively identified compounds in the samples.

### 2.8 Statistical analysis

All statistical analyses were performed using the R Studio Software ver. 2022.07.02 Build 576. The differences in the growth and the different parameters were analysed by ANOVA at p-values<0.05. The level of significance was established at p<0.05. The results were expressed as means ± SE of the mean.

## 3 Results

### 3.1 Callus induction from floral buds

**Figure 1:**
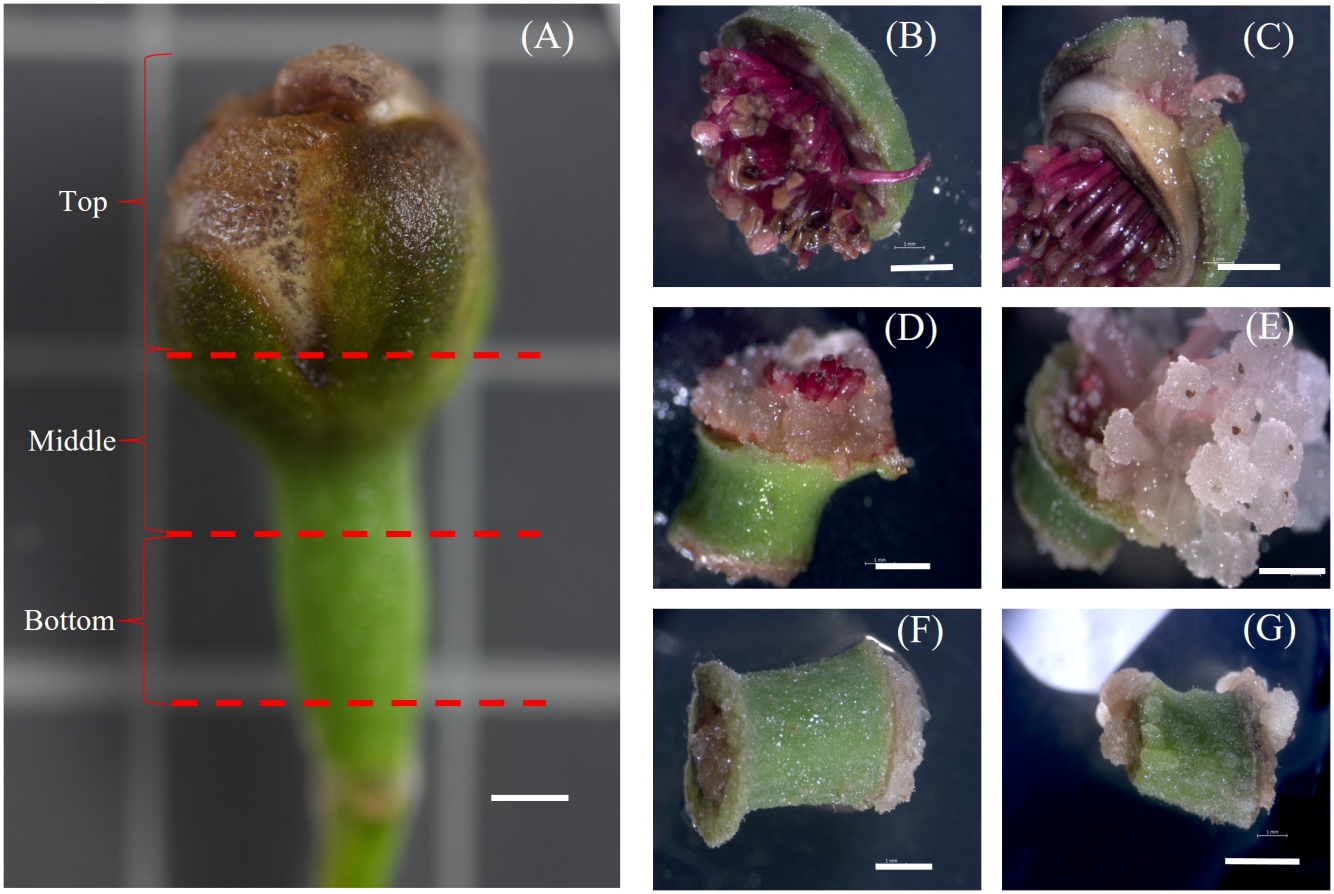
Callus induction from different regions of Feijoa floral bud. **(A)**: Floral Bud showing the different regions of explants. **(B, C)**: Top region; **(D, E)**: Middle region; **(F, G)**: Bottom region; Scale bar = 2mm.

**Figure 2:**
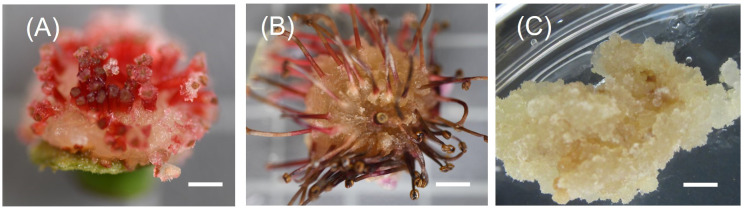
Induction of friable callus. **(A)**: Callus induce after one week in culture from the base of stamens; **(B)**: Callus growth after 3 weeks in culture; **(C)**: Friable callus 8 weeks old maintained by subculturing; Scale bar = 5mm.

**Figure 3:**
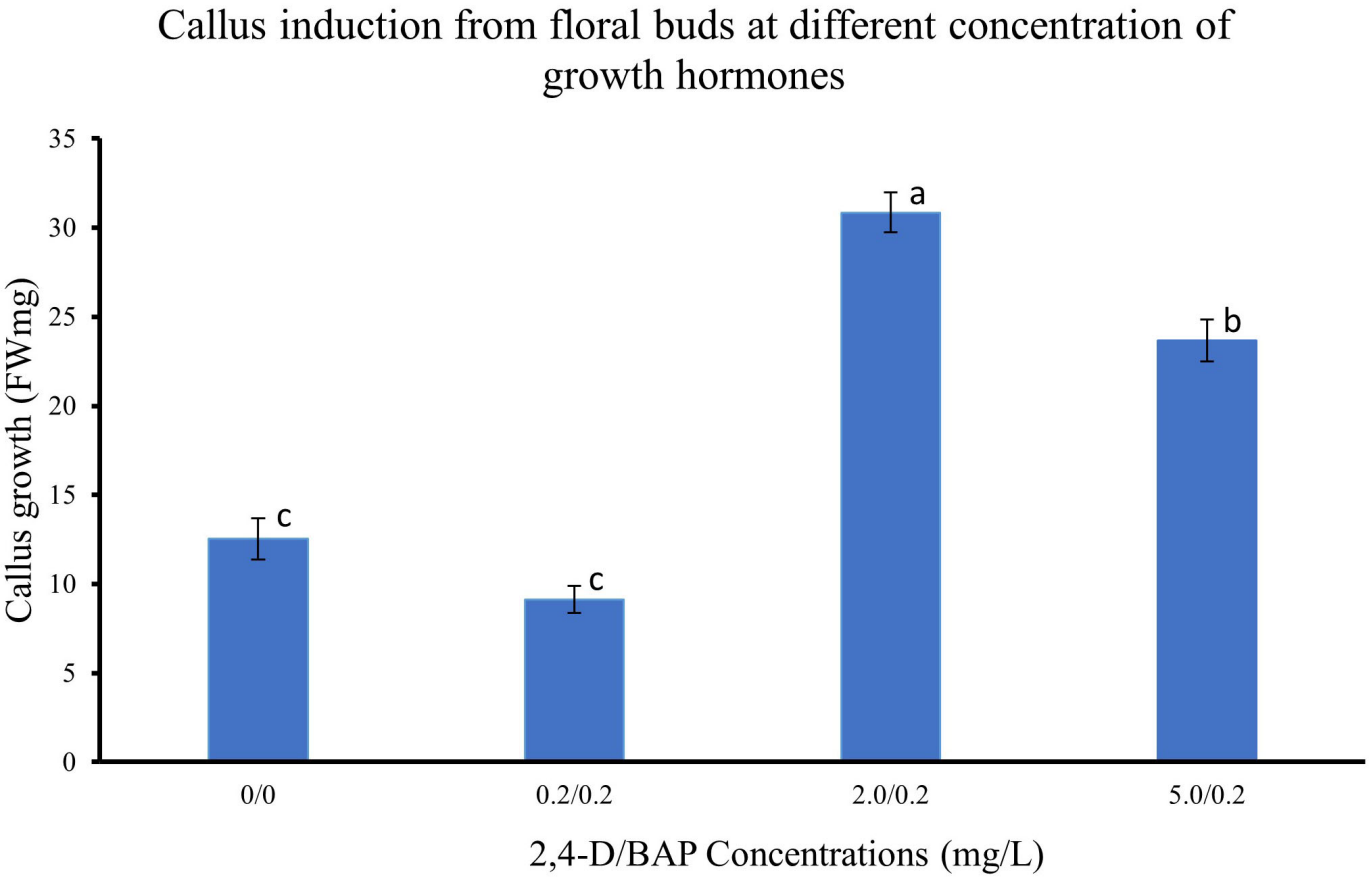
Callus induction from floral buds at different concentration of 2,4-D. Values are means from three replications. Error bars represent standard error. Different letters on each bar represent statistically significant differences at P ≤ 0.05 as determined by LSD test.

The different regions of the floral buds, viz., top, middle and bottom regions, responded differently to the different concentrations of 2,4-D with BAP concentration constant at 0.2 mg/L as seen in the callus induction pattern (**Figure 1**). The top region of the bud, which mostly consisted of whorls of sepals, petals and stamens, resulted in lesser callus induction. However, the middle region, which had the bases of stamens connected with the ovary, had the most callus induction (**Figures 1D, E**, **2A–C**). The lower region also did not reciprocate as well as compared to the top two regions (**Figures 1F, G**). Also, differences were observed in the texture of the callus from the different regions. The Callus originating from the basal region of the floral buds was more rigid, while the callus originating from the middle and top regions of the floral buds was more friable and translucent than the callus originating from the basal region. Callus initiation response was observed after 10 days in culture in all regions of the floral buds. When the data on callus induction at different concentrations of 2,4-D was analysed, it was observed that the callus could be induced across the range of concentrations tested. Callus induction was seen on MS medium even without PGR (**Figure 3**). However, callus induction was most prominent, with 31 mg FW and 24 mg FW observed at 2 mg/L and 5 mg/L 2,4-D concentration, respectively with BAP concentration at 0.2 mg/L (**Figure 3**).

### 3.2 Initiation of cell suspension cultures

**Figure 4:**
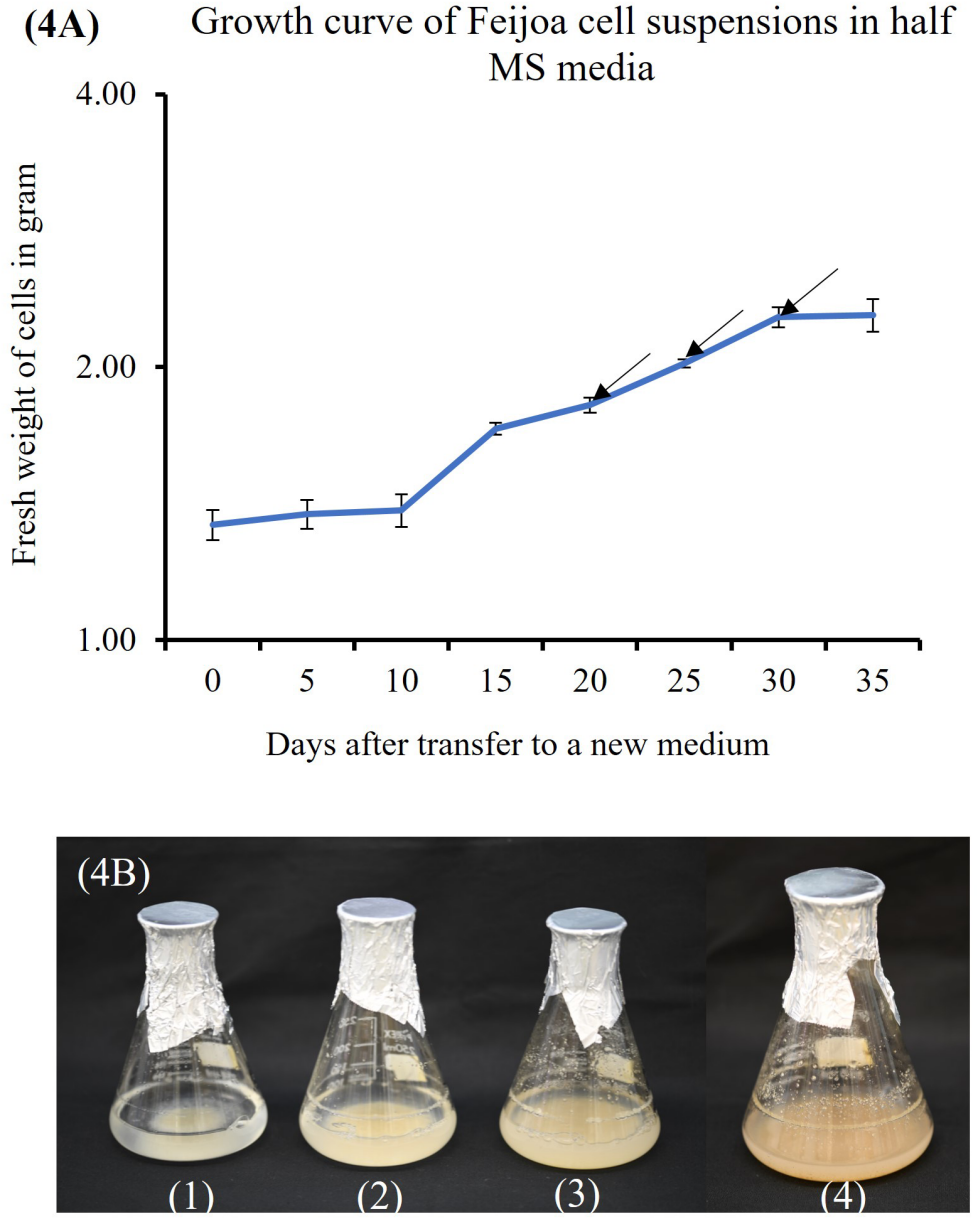
Feijoa cell suspension growth curve and the different phases as observed in flasks during the different phases of growth curve. **(A)** Growth curve of Feijoa cells in half MS media. Arrows indicate treatment of cell suspensions with different concentration of MeJA. Results of phytochemicals analysed are shown in **Table 1**. The growth curve was investigated using half strength MS media. Values are means from three replications. Error bars represent standard error. **(B)** Feijoa cell suspensions at different phases of the growth curve. 1: Lag phase, 2&3: Linear phase, 4: Stationary phase.

Cell suspension cultures of feijoa using a half-strength MS media showed a sigmoidal growth curve (**Figure 4A**), which started with the lag phase (0-10 days) (**Figure 4B**1) followed by the linear phase (10-30days) (**Figure 4B**2, **B**3) and ended with the stationary phase (after 30 days) (**Figure 4B**4). After 3 weeks in culture, the colour of cell suspension changed to light brown and, after 4 weeks, turned dark brown, indicating an increased level of phenol synthesis and also resulting in the death of cells.

### 3.3 Effect of MS media strength on the growth of feijoa cell suspensions

**Figure 5:**
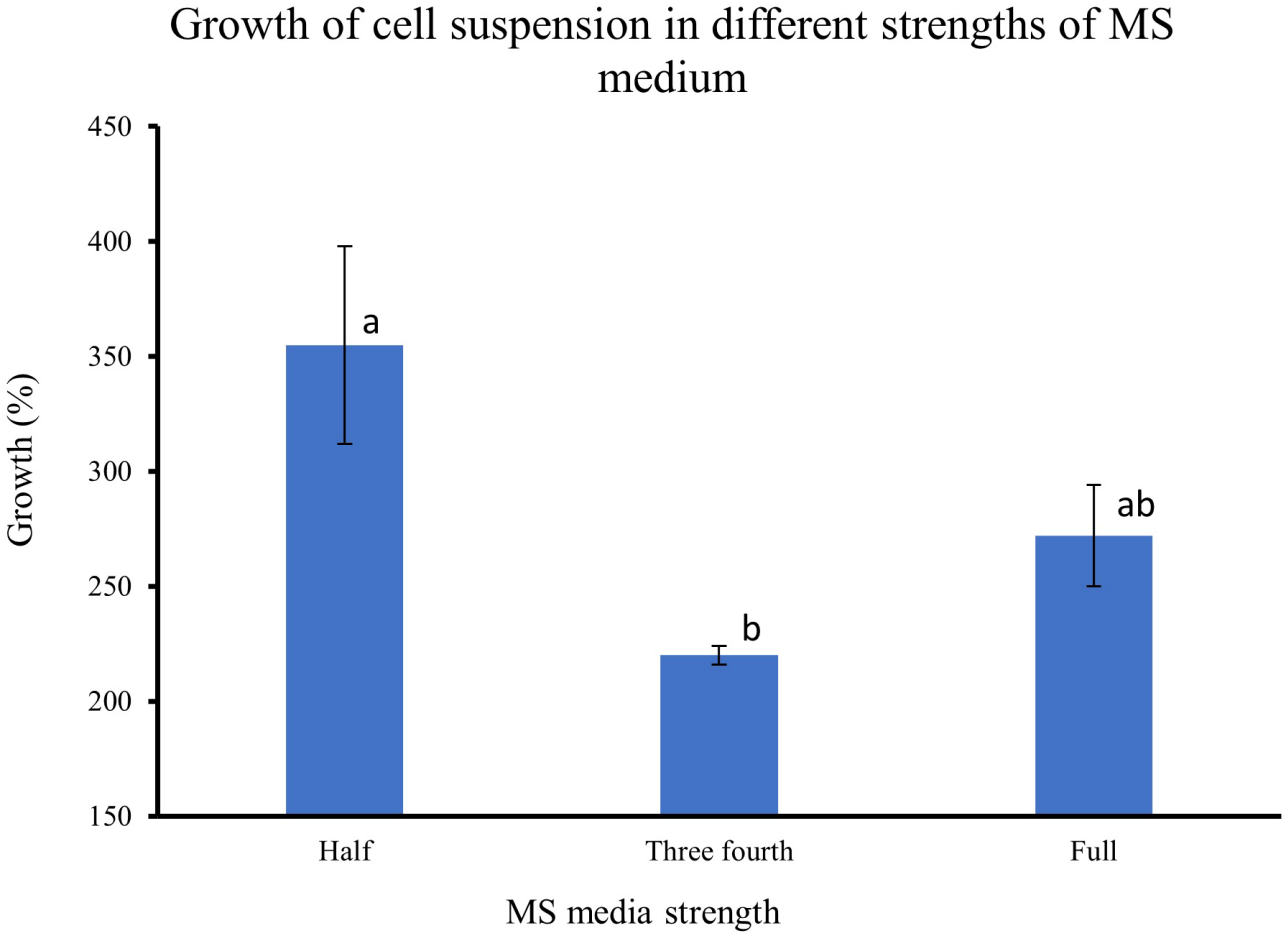
Effect of different strengths of MS medium on the growth of cell suspensions. The initial fresh weight of the inoculum was 3g/100ml media. Values are means from three replications. Error bars represent standard error. Different letters on each bar represent statistically significant differences at P ≤ 0.05 as determined by LSD test.

The results from feijoa cell suspension cultures at different strengths of MS medium showed variability in the growth of the cells. The growth of cell suspension at half-strength MS medium was markedly higher (355%) than the growth of cell suspensions at three-fourths (220%) or full-strength MS medium (272%) (**Figure 5**). However, the difference in the growth of the cell suspension between three-fourth and full-strength MS medium was not significantly different.

### 3.4 Effect of sucrose concentrations on the growth of feijoa cell suspensions

**Figure 6:**
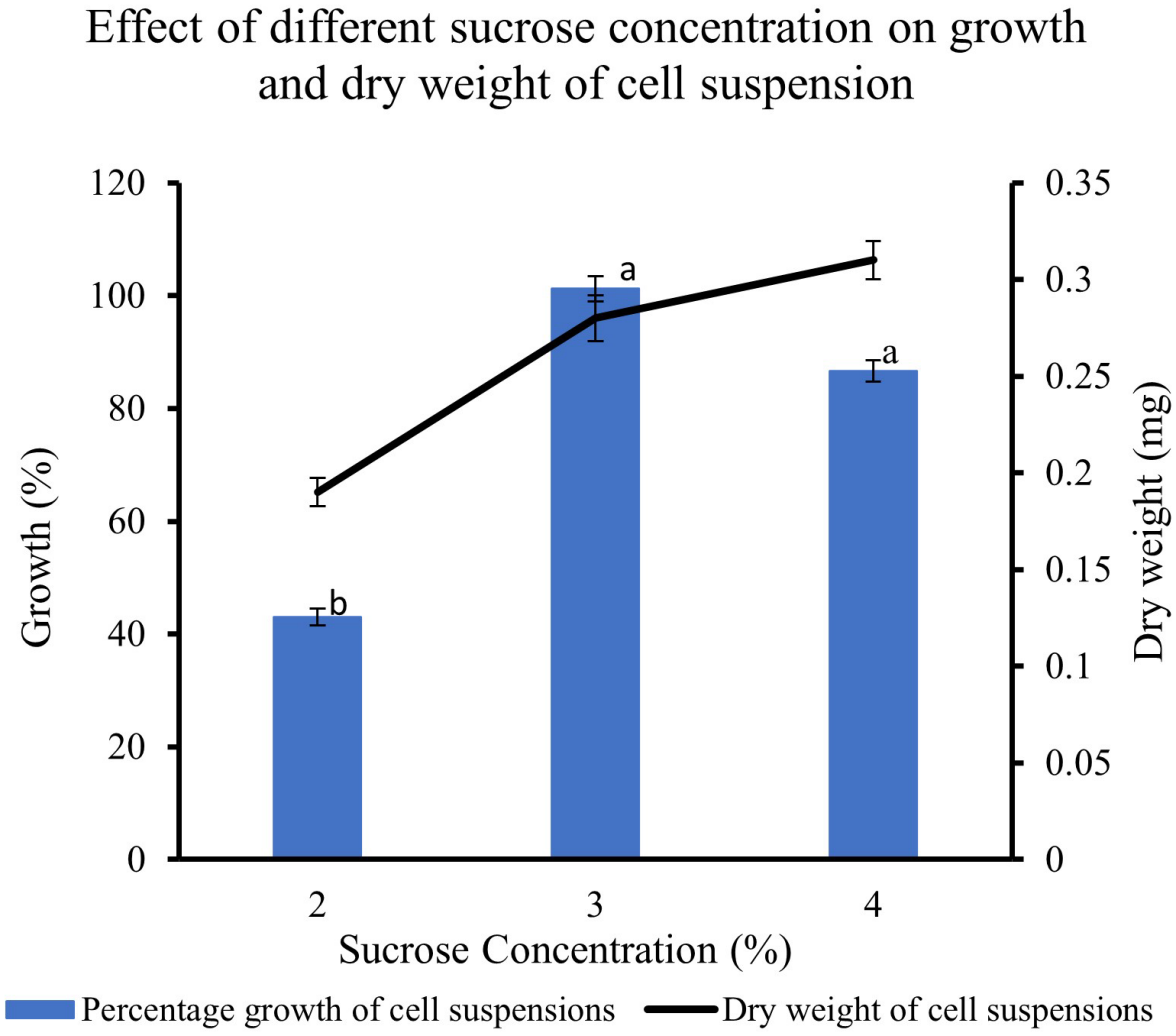
Effect of different sucrose concentration on the growth and dry weight of cell suspensions. Values are means from three replications. Error bars represent standard error. Different letters on each bar represent statistically significant differences at P ≤ 0.05 as determined by LSD test.

The feijoa cell suspensions cultured in full-strength MS medium at 3% and 4% sucrose concentration showed a much higher percentage growth (101% and 87%, respectively) than the 43% growth achieved when cell suspension was cultured at 2% sucrose. The growth achieved at 4% sucrose tended to decrease; however, it was not significant. The dry weight of the cell suspension also increased with the concentration of sucrose (**Figure 6**).

### 3.5 Effect of inoculum density on the growth of feijoa cell suspensions

**Figure 7:**
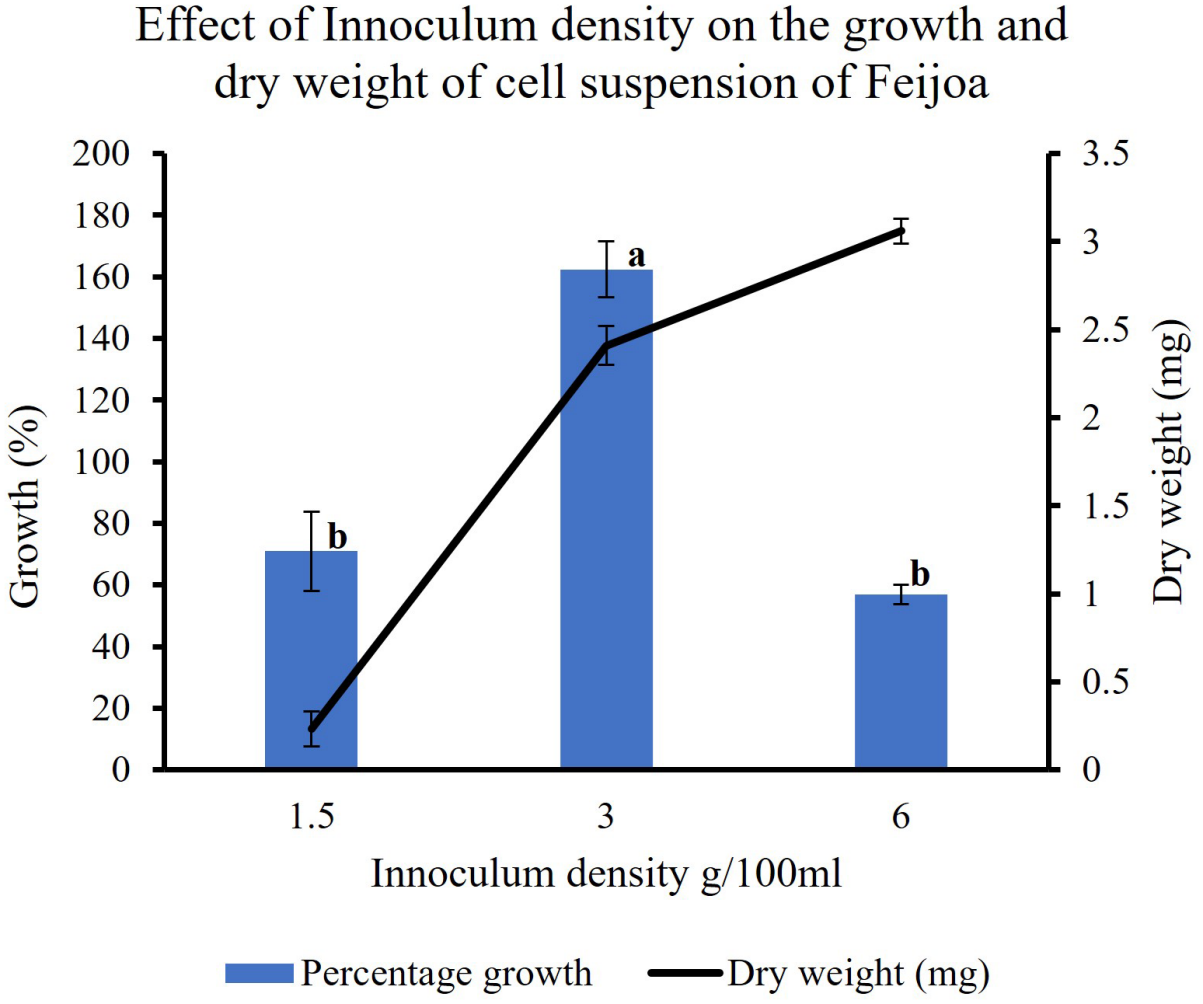
Effect of inoculum densities on the growth of cell suspensions. Values are means from three replications. Error bars represent standard error. Different letters on each bar represent statistically significant differences at P ≤ 0.05 as determined by LSD test.

In feijoa cell suspension cultured at different densities, using the basal MS medium supplemented with sucrose 3% and 2,4-D and BAP at 2 mg/L and 0.2 mg/L concentration, respectively, the highest growth (162%) was observed at a density of 3 g/100ml of medium (**Figure 7**). A significantly lower percentage growth, viz., 71% and 57%, was observed at 1.5 g/100 mL and 6 g/100 mL densities, respectively. This indicates that inoculum density is a key parameter when considering the growth of feijoa cell suspensions.

### 3.6 Effect of 2,4-D concentrations on the growth of feijoa cell suspensions

**Figure 8:**
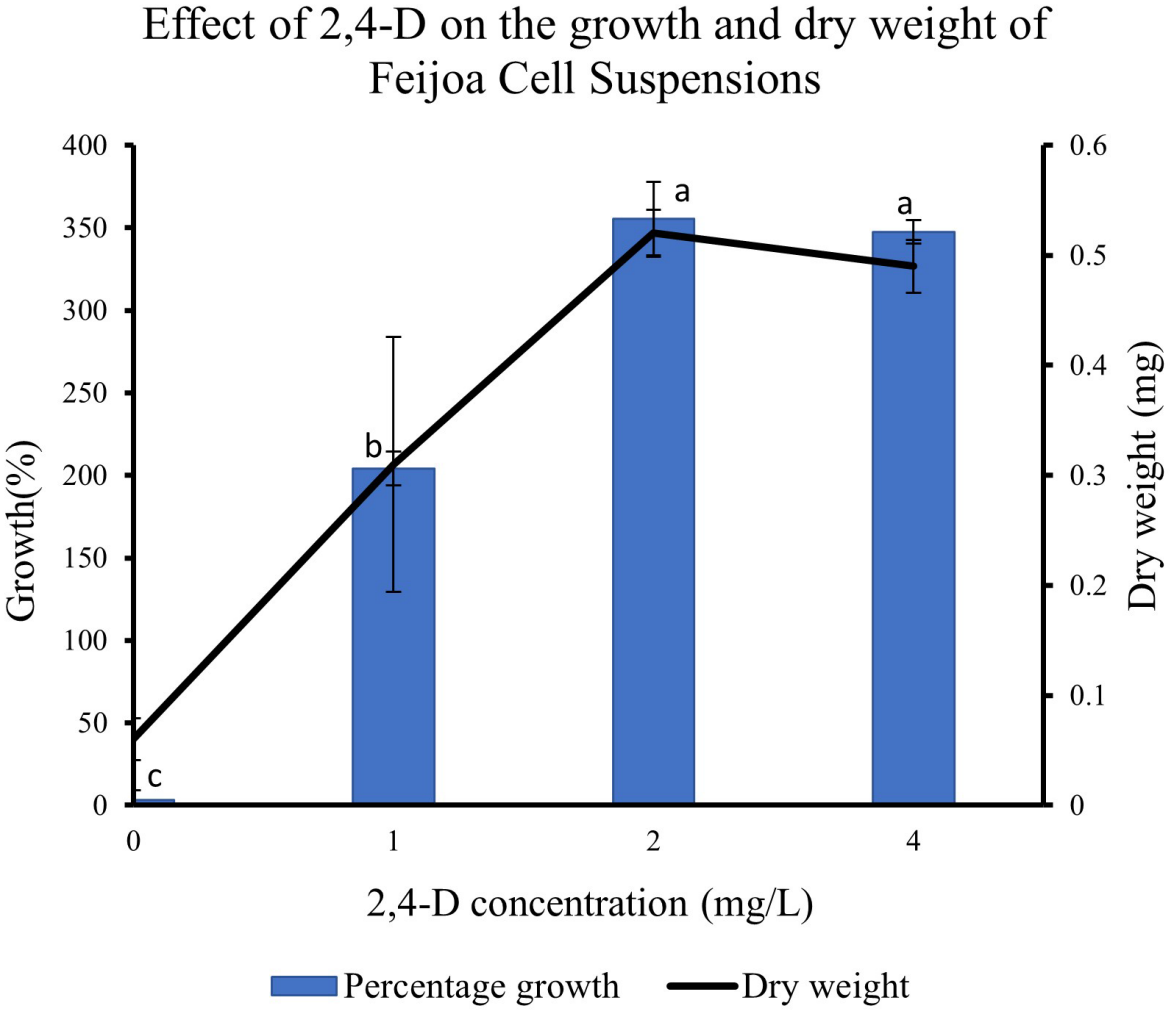
Effect of 2,4-D concentration on growth of Feijoa cell suspensions. Values are means from three replications. Error bars represent standard error. Different letters on each bar represent statistically significant differences at P ≤ 0.05 as determined by LSD test.

Supplementing the MS media with 2,4-D had a pronounced effect on the growth of cell suspension (**Figure 8**). When cell suspensions were cultured in MS media without 2,4-D and BAP, a minimal percentage growth of 0.31% in cell suspension was recorded. The highest percentage growth of 355% and 347% was recorded with 2,4-D concentrations of 2 mg/L and 4 mg/L, respectively, with BAP constants at 0.2 mg/L. However, the growth decreased by almost 150% when cell suspension was cultured at 2 mg/L 2,4-D and 0.2 mg/L BAP concentration. The results indicate that the cell suspension growth depends on exogenous source of auxin in the medium and could vary with the concentrations used. The dry weight of cell suspensions also increased with the concentration of 2,4-D with 0.52 mg and 0.49 mg recorded at 2 mg/L and 4 mg/L, respectively compared to 0.31 mg recorded at 1 mg/L.

### 3.7 Effect of different sugars on the growth of feijoa cell suspensions

**Figure 9:**
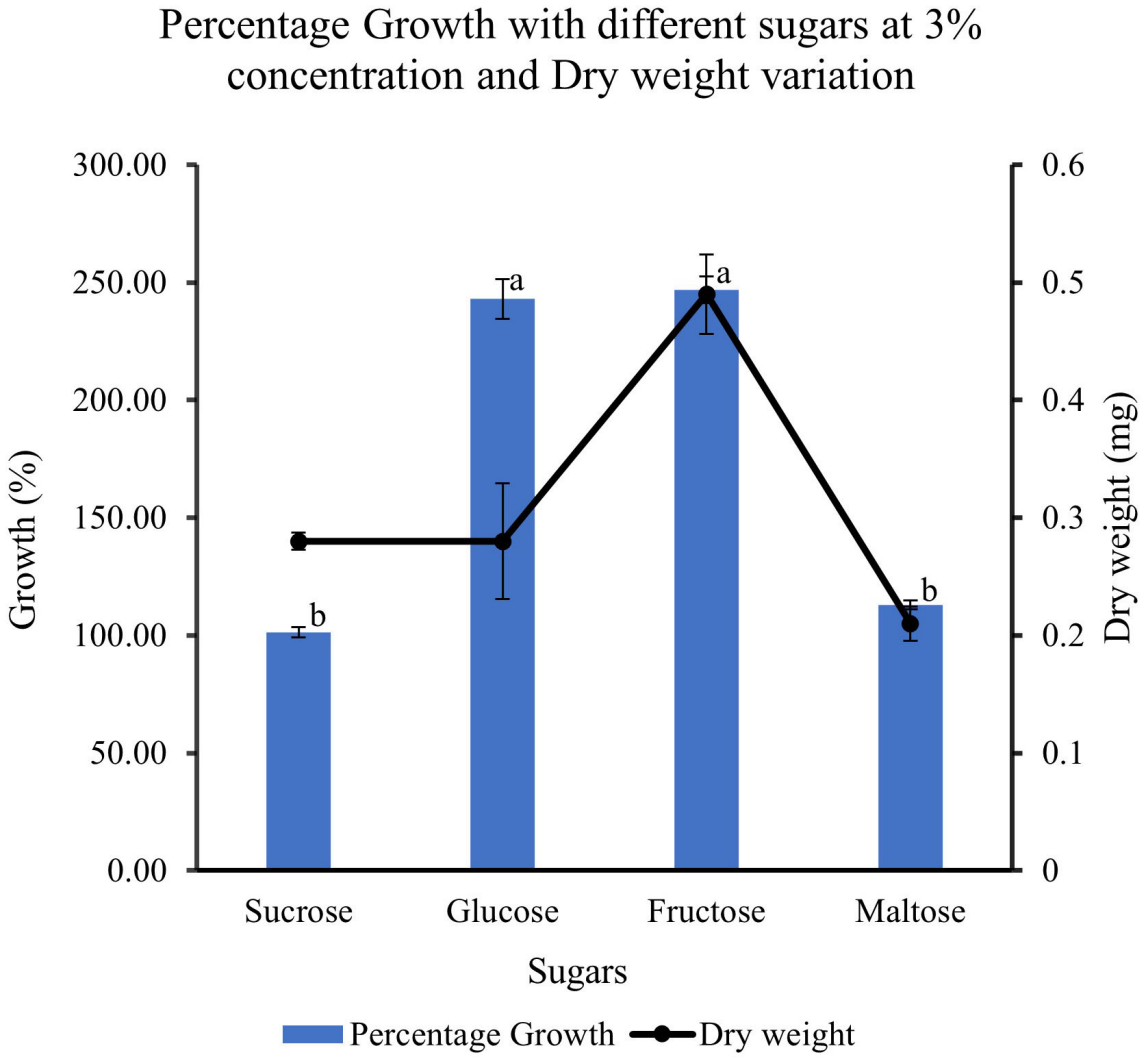
Effect of different sugar sources on the growth and dry weight of cell suspensions. Values are means from three replications. Error bars represent standard error. Different letters on each bar represent statistically significant differences at P ≤ 0.05 as determined by LSD test.

When feijoa cell suspension was cultured in MS medium supplemented with different sugars, the response observed on the growth of cell suspension was varied (**Figure 9**). The highest percentage growth of 243% and 247% was observed in cell suspension cultured in MS medium supplemented with 3% glucose and in MS medium with 3% fructose, respectively. However, there was a marked decrease of almost 150% in the percentage growth of cell suspensions cultured in MS medium supplemented with 3% sucrose (101%) and cell suspensions cultured in 3% maltose (113%). The results indicate that there might be preferences for sugar sources for feijoa cell growth. The dry weight of cell suspension also showed variability, with the highest accumulation of dry weight when fructose was used.

**Figure 10:**
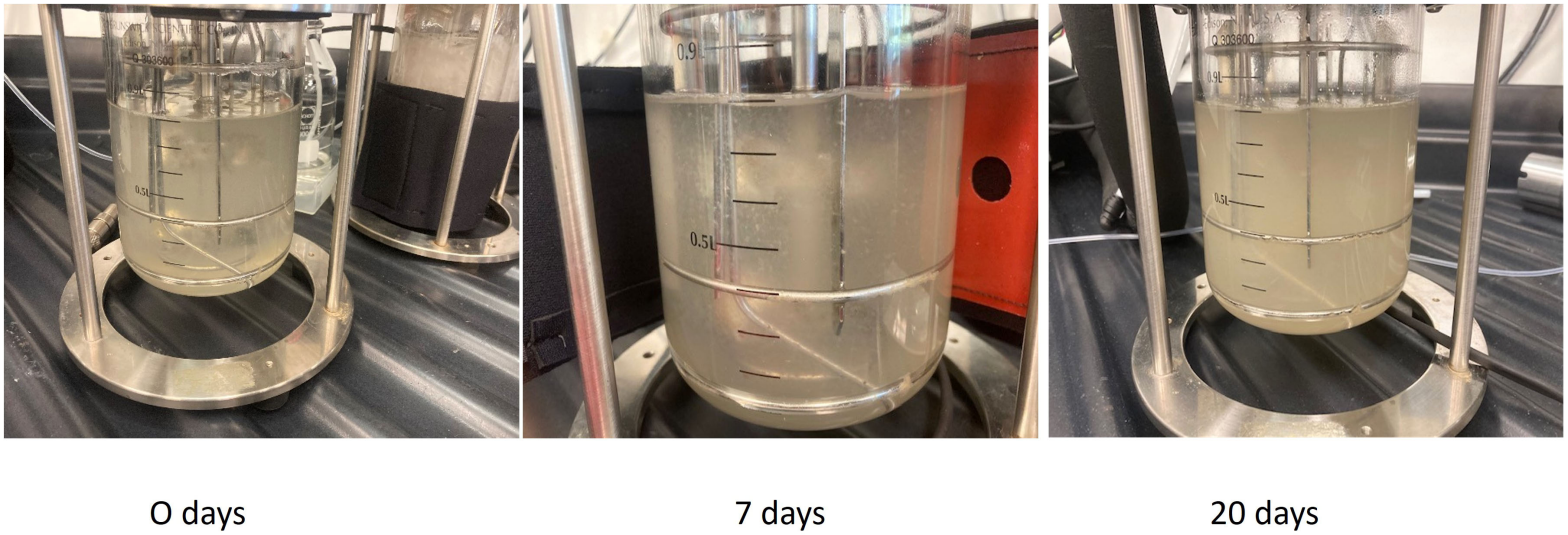
Culture of Feijoa cell suspensions in 1L bioreactors. The cultures were initiated with an initial inoculum of 30 gm in half MS media supplemented with 2,4-D and BAP at 2 and 0.2 mg/L concentration respectively.

In addition to the above experiments, a one litre bioreactor was set up in the dark at room temperature as a trial experiment to generate biomass. The trial was conducted throughout the period of the growth cycle for 30 days. The initial inoculum weight at the start of the experiment was measured as 30g. The final weight of the cell mass at the end of the growth cycle after 30 days was 75g. The cell biomass harvested was more than double the initial inoculum weight (**Figure 10**, data not shown). However, detailed investigations focused on optimising the bioreactor setup, and operationality will be part of further studies.

### 3.8 Phytochemical UPLC- QTOF MS analysis results in elicitation experiment

Phytochemical characterisation results on cell suspensions elicited with MeJA experiment and floral buds (section 2.5 in methods above) are herein reported. A large range of compounds were detected in the samples at different time points and days. Among these compounds, there were secondary metabolites (flavonoids and flavonoid-glucosides, phenylpropanoids, amino acid related compounds, coumarins, etc.) and plant hormones which could be of interest for their potential use as therapeutics or in skin and personal care products. These compounds are reported in **Table 1** (including the retention time (RT), score match with mass spectrum library, mass to charge ratio (m/z) and ion species identified). In **Supplementary Tables S3 (A–C)**, each compound identified in all samples is reported. Summarised in **Table 2** is the number of compounds accumulated during the timeline of the experiment, at different concentrations of MeJA and on different days after elicitation. In general, the number of compounds increased with the elicitation, with the highest number of compounds detected using 50µM MeJA and measured at 20 days. Floral bud extracts were analysed, and the classification of identified compounds is also reported in **Tables 1**, **2**, and **Supplementary Table S3D**. When comparing the numbers of compounds identified in cell suspensions with floral buds, cell suspensions reported higher amounts than floral buds (average of all days).

**Table 1:** Putatively identified compounds analysed by UPLC - QTOF MS in tissue culture suspensions and floral buds.

Compound	Score	m/z	RT	Ion Species
(-)-Epicatechin gallate	99.87	441.0806	9.17	(M-H)-
(-)-Epigallocatechin gallate	98.72	457.0762	8.75	(M-H)-
(+)-Artemisinate	99.94	279.1607	11.57	(M+COOH)-
(+)-Secoisolariciresinol	99.58	407.1175	10.10	(M+COOH)-
(+)-Taxifolin	99.19	303.0501	9.47	(M-H)-
(2R,3S,4S)-Leucocyanidin	99.94	305.0670	17.85	(M-H)-
(2S,4As,6aS,6bR,10S,12aS)-10-hydroxy-2-(hydroxymethyl)-2,4a,6a,6b,9,9,12a-heptamethyl-3,4,5,6,6a,7,8,8a,10,11,12,14b-dodecahydro-1H-picen-13-one	99.94	455.3523	14.73	(M-H)-
(E)-6’-Hydroxyferulate	98.24	209.0449	8.14	(M-H)-
(S)-laudanosine	99.94	356.1861	11.27	(M-H)-
1,2,6-Trigalloylglucose	95.91	635.0881	8.64	(M-H)-
1,2-Di-O-sinapoyl-beta-D-glucose	98.82	591.1685	9.89	(M-H)-
1,6-Bis-O-galloyl-β-D-glucose	98.82	483.0771	8.22	(M-H)-
16-α-Hydroxygypsogenate	92.44	501.3211	11.30	(M+COOH)-
1-O-Vanilloyl-β-D-glucose	99.87	329.0868	8.14	(M-H)-
2-Coumarate	99.77	163.0395	9.00	(M-H)-
2’-Hydroxy 3,6,7,3’,4’-pentamethylquercetagetin	99.71	449.1075	9.25	(M+COOH)-
2-Hydroxy-2,3-dihydrogenistein-7-olate	99.81	287.0555	9.92	(M-H)-
2-Methoxycarbonylphenyl b-D-glucopyranoside	99.91	359.0973	8.17	(M+COOH)-
3,5-Di-C-glucosyl-2,4,4’,6-tetrahydroxydibenzoylmethane	98.29	611.1569	8.88	(M-H)-
3’,5’-Di-C-glucosylphloretin	99.76	643.1876	7.92	(M+COOH)-
3,5-Didehydroshikimate	99.40	169.0137	4.49	(M-H)-
3,6,7,2’,4’-Pentamethylquercetagetin 3’-O-β-D-glucoside	97.18	611.1577	8.88	(M+COOH)-
3-β-Hydroxyglycyrrhetinate	99.93	515.3365	12.57	(M+COOH)-
3-Hydroxy-5-methoxybiphenyl	99.87	245.0813	8.43	(M+COOH)-
3-Hydroxy-beta-ionone	99.38	253.1439	11.29	(M+COOH)-
3-Isobutanoyl-3’,4-di(isovaleryl)sucrose	97.90	579.2644	8.90	(M+COOH)-
3-Lauryl-3’-isobutanoyl-4-(isovaleryl)sucrose	99.36	723.3783	11.96	(M+COOH)-
3’’-O-(4-Hydroxycinnamoyl)astragalin	99.93	593.1282	8.20	(M-H)-
4-(Isobutanoyl)sucrose	97.31	411.1492	8.44	(M-H)-
4α-Carboxy-5alpha-cholesta-7,24-dien-3beta-ol	99.90	473.3270	11.77	(M+COOH)-
4α-Carboxy-ergosta-7,24(241)-dien-3beta-ol	99.90	487.3419	11.77	(M+COOH)-
4α-Formyl-ergosta-7,24(241)-dien-3beta-ol	99.97	471.3469	13.30	(M+COOH)-
4α-Hydroxymethyl-4beta-methyl-5alpha-cholesta-8,24-dien-3beta-ol	99.35	473.3623	12.62	(M+COOH)-
4-Coumaroylshikimate	99.07	319.0813	8.77	(M-H)-
4’-Demethylepipodophyllotoxin	98.24	445.1131	8.84	(M+COOH)-
5-O-Caffeoylshikimate	98.87	335.0763	8.46	(M-H)-
6-O-Galloyl-β-D-glucose	98.75	331.0661	4.48	(M-H)-
9,10,18-Trihydroxystearate	93.48	331.2479	11.40	(M-H)-
9’-Hoaba	89.24	279.121	8.90	(M-H)-
Abscisic alcohol	99.93	249.1488	13.85	(M-H)-
Alizarin	90.40	285.0401	9.35	(M+COOH)-
α-3’,4’-Anhydrovinblastine	96.20	791.3994	10.22	(M-H)-
α-3’,4’-Anhydrovinblastine radical	85.69	789.3888	10.57	(M-H)-
Arctigenin	100.00	371.1512	15.24	(M-H)-
Artemisinin	97.21	281.1391	9.48	(M-H)-
Astilbin (Taxifolin 3-O-rhamnoside)	99.71	449.1075	9.27	(M-H)-
Avenastenone	99.94	455.3523	14.73	(M+COOH)-
Baicalin	99.44	491.0821	8.90	(M+COOH)-
Benzoyl-β-D-glucoside	99.87	329.0868	8.14	(M+COOH)-
Bergapten	84.80	215.0341	8.72	(M+COOH)-
Biliverdine	86.60	581.2372	9.09	(M-H)-
C19-Gibberellin skeleton	80.29	361.1627	11.56	(M+COOH)-
Catechin	99.75	289.0711	8.50	(M-H)-
Chalconaringenin 4’-glucoside	97.12	433.1125	9.62	(M-H)-
*cis*-Zeatin	93.36	218.1026	7.65	(M-H)-
*cis*-zeatin-7-N-glucoside	93.43	380.1588	14.68	(M-H)-
Combretol	97.12	433.1125	9.62	(M+COOH)-
Coproporphyrinogen III	94.56	659.3132	14.72	(M-H)-
Cyanidin 3-O-(6’-O-malyl-β-D-glucoside)	93.81	609.1142	9.54	(M-H)-
Cyanidin 3-O- β -D-caffeoylglucoside	99.18	609.1226	7.84	(M-H)-
Daphnetin	99.90	177.0188	9.60	(M-H)-
Dhurrin	97.10	310.09	9.39	(M-H)-
Dicrocin	99.29	651.2668	9.44	(M+COOH)-
Dihydroprecondylocarpine acetate	94.12	441.202	15.39	(M-H)-
Diphyllin	99.10	425.0863	8.02	(M+COOH)-
Eugenyl-GXG	94.67	665.2251	8.58	(M+COOH)-
Gaultherin	99.90	491.1391	8.02	(M-H)-
Gentisate 5-O-β-D-xylopyranoside	98.79	331.0659	4.48	(M+COOH)-
Geranyl 6-O-β-D-xylopyranosyl-β-D-glucopyranoside	99.62	447.2233	10.17	(M-H)-
Gingerol	99.89	293.175	11.57	(M-H)-
Ginsenoside C-K	99.25	621.4354	17.06	(M-H)-
Ginsenoside rb1	88.19	1153.601	11.24	(M-H)-
Glycyrrhetinic aldehyde	99.53	453.3368	14.32	(M+COOH)-
Gypsogenate	99.38	431.3326	12.12	(M+COOH)-
Hederagenin	99.97	471.3469	13.30	(M-H)-
Heptaketide pyrone	99.13	321.0605	8.36	(M+COOH)-
Jasmonate	99.75	209.1167	10.93	(M-H)-
Leukoefdin	97.71	321.0606	8.07	(M-H)-
Linamarin	99.28	246.0975	8.09	(M-H)-
Lotaustralin	98.37	260.1137	8.75	(M-H)-
Macarpine	97.17	436.1042	7.96	(M+COOH)-
Mermesin	99.87	245.0813	8.43	(M-H)-
Methylsalicyl-GXG	95.97	653.1913	7.55	(M+COOH)-
Naringenin chalcone	99.86	271.0607	10.63	(M-H)-
Neolinustatin	97.12	468.1711	8.10	(M+COOH)-
Nothofagin	99.94	435.1284	9.21	(M-H)-
Olivetol	99.81	225.113	9.34	(M+COOH)-
Phloretin	99.07	273.0754	8.91	(M-H)-
Pinostrobin	99.78	315.0864	9.21	(M+COOH)-
Procyanidin B2	99.92	577.1338	8.22	(M-H)-
Quercetagetin 7-O-glucoside	99.72	479.0823	7.87	(M-H)-
Quercetin	86.58	301.0346	9.55	(M-H)-
Rishitin	99.91	221.1539	11.57	(M-H)-
Salicyl-6-hydroxy-2-cyclohexene-on-oyl	98.58	261.0757	7.87	(M-H)-
Sasanquin	95.95	503.1737	10.56	(M+COOH)-
Sissotrin	99.09	445.1131	8.84	(M-H)-
Tetraketide pyrone	99.07	223.0975	8.16	(M-H)-
*trans*-Tuberonic acid glucoside	97.97	387.1653	8.41	(M-H)-
Tuberonate	99.81	225.113	9.34	(M-H)-
Vanillate	98.92	167.0343	9.34	(M-H)-
Vestitone	97.70	331.0824	8.40	(M+COOH)-
Vernolic acid	99.05	341.2331	11.01	(M+COOH)-
Vincristine	98.79	823.3891	9.60	(M-H)-

**Table 2:** Number of compounds identified after MeJA elicitation (50 µM *vs* 100 µM *vs* ctrl), at different time points (0-72 hours) after 20, 25 and 30 days compared with floral bud content.

Timeline (Hours)	0			6			12			24			48			72			Floral buds
Methyl Jasmonate concentration (µM)	50	100	ctrl	50	100	ctrl	50	100	ctrl	50	100	ctrl	50	100	ctrl	50	100	ctrl	NA
20 days	70	63	50	74	76	50	68	71	39	81	78	27	79	76	40	80	74	36	25
25 days	69	59	59	77	54	59	76	64	60	78	62	64	78	60	55	77	57	59	25
30 days	56	54	57	59	68	57	56	67	54	61	71	58	70	72	56	70	68	54	25

**Figure 11:**
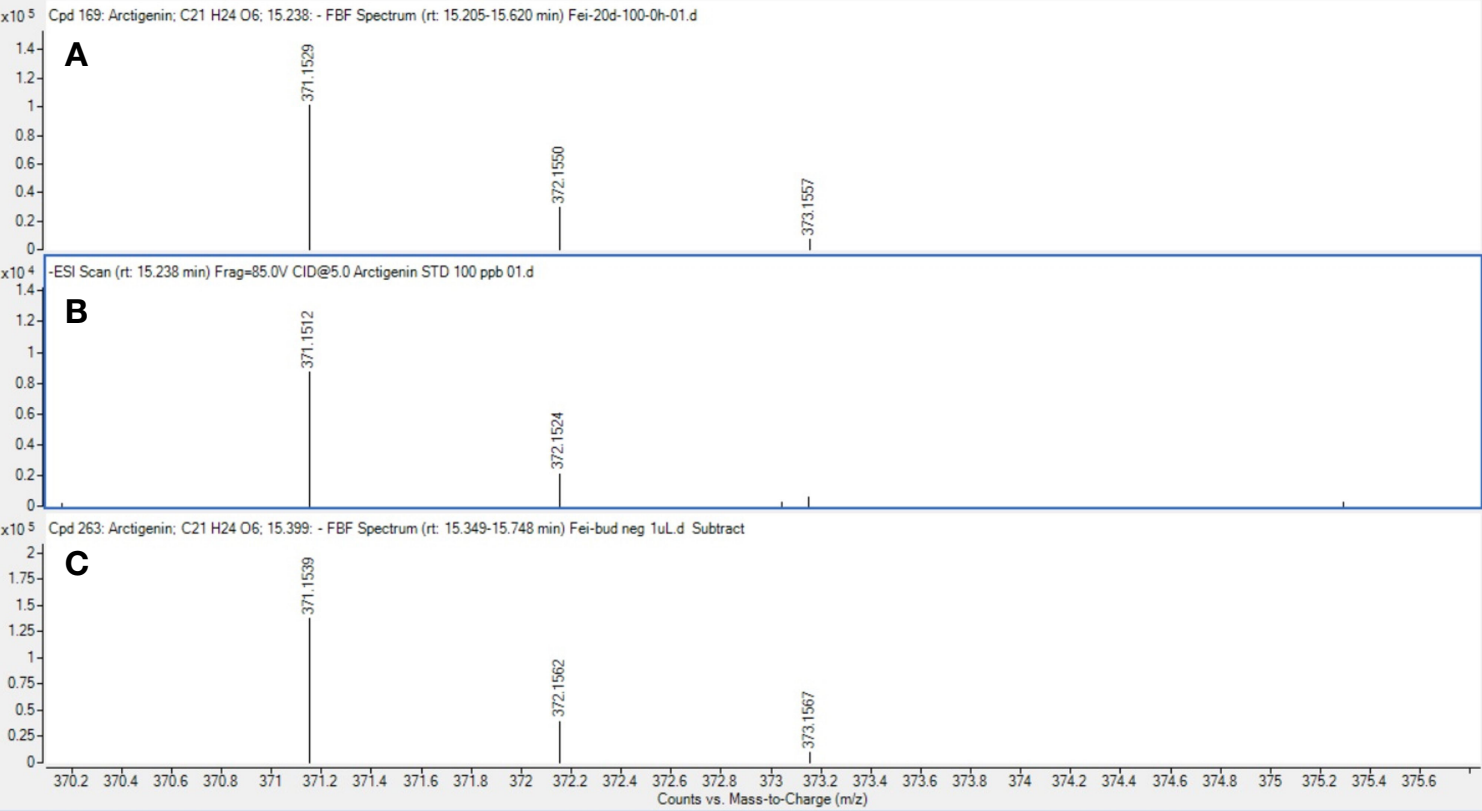
Mass spectrum of arctigenin detected in samples. Mass spectrum of arctigenin (m/z 371.1512 at 15.24 min) in cell suspension cultures **(A)**, commercial standard reference **(B)** and floral buds **(C)**.

As an example of a compound of interest, the confirmation of the presence of arctigenin eluting at 15.24 minutes (371.1512 m/z) in all the cell suspensions and floral bud extracts is reported (**Figure 11**). Arctigenin identity assignment was confirmed with the use of a commercially available standard which was analysed using the same methodology as samples. Relative quantification of the arctigenin content was performed using a single external standard concentration resulting in a relative amount of arctigenin in the samples in the range of 4-8 ppm (parts per million) extracted from cell suspension and floral bud (data not shown). A higher amount of arctigenin was detected in the floral buds when compared to samples treated with 50 and 100 µM MeJA (data not shown).

## 4 Discussion

Various studies have shown that feijoa plants produce different compounds that can benefit human well-being. Weston (2010) published a comprehensive review of the application of feijoa bioactive compounds for various purposes. Feijoa extracts have been tested for their efficacy against various human ailments. More recently, bioactive compounds were identified and assessed from feijoa callus and cell suspensions for their anticoagulant properties, which resulted in decreased platelet aggregation (Amer et al., 2023). The source of these extracts has so far been the fruit or the fruit peel. Cell suspension cultures offer an alternative method to produce high-value compounds in large quantities and high purity in contained environments where biotic and abiotic factors responsible for the production of these compounds can be monitored and regulated. *In vitro* cultures, such as cell suspensions, provide a continuous and reliable source of bioactive compounds making the process standardised and economically sustainable (Davies and Deroles, 2014). However, biomass accumulation and synthesis of bioactive compounds by the cultured cells is a two-stage process as proposed by Murthy et al. (Murthy et al., 2014), which involves: stage 1) the establishment of a cell suspension system and stage 2) the elicitation of the bioactive compounds, which are controlled by various factors.

Friable callus induction is a prerequisite to generate cell suspensions. Different plant groups respond differently to the callus induction process, which depends on many factors, such as types and concentrations of auxins and cytokinins, the tissue and the physiological state of the tissue. During earlier research studies on feijoa, where application of callus has been majorly restricted to achieve somatic embryogenesis, callus had been induced from stamen filaments, petals and ovaries (Stefanello et al., 2005), whole intact cotyledonary zygotic embryos (Reis et al., 2008), fruit pulp (Verardo et al., 2019) and leaves (Amer et al., 2023). In this study, we used floral buds without separating stamen filaments, petals, or ovaries but used the regions of floral buds as explants (**Figure 1**). Callus was induced in 10 days’ time from all regions of the floral buds, which is similar to the results obtained by Stefanello et al. (2005). As highlighted in many other studies the auxin 2,4-D is one of the key exogenous auxins to evoke a response from explants for callus induction. Menbari et al. (2021) used 2,4-D and BA to induce callus from Granny Smith apples, which were later used to generate cell suspension cultures. Soleimani et al. (2012) also reported the highest callus induction from *Arctium lappa* using 2,4-D and BAP. Our study results support those findings. However, we also observed callus induction in our control treatment without 2,4-D and BAP (**Figure 3**). This could be attributed to the highly active metabolic state of the explants, which responded to the wounding process. The callus in our control treatment could be classified as a wound-induced callus, which can be derived from various cell types such as vascular-associated cells, cortical cells and pith cells (Ikeuchi et al., 2013). This response of callus induction without any exogenous hormones could have also been influenced by the transitioning phase of the floral bud, where the stage includes a complex system of factors which interact and involve various biochemical signals, including plant hormones, polyamines, gibberellic acids and cytokinins (Meijón et al., 2011).

Friable callus was selected for initiation of suspension cultures. The friable property of callus helps initiate suspension cultures when cultured in a liquid medium, which helps easy dispersion and growth of the cells in a liquid medium when agitated. The biomass accumulation in the medium depends on multiple factors such as the salt concentrations, inoculum density and the type and concentration of the sugars used in the medium (Murthy et al., 2014). When we cultured the cell suspension using different strengths of MS medium to understand the requirement of salt concentration for optimal growth of biomass, the highest biomass growth was achieved using half-strength MS medium as compared to three-fourth and full-strength medium (**Figure 7**). The salt requirements in the medium have been shown to vary in cells from different plants (Wu et al., 2006; Nagella et al., 2011; Naik et al., 2017). It has also been reported that during *Panax ginseng* root biomass production, three-fourth strength MS medium produced better results (Sivakumar et al., 2005). Improving the growth and biosynthetic capacity of the *in vitro* generated plant cell mass by developing optimal culture conditions through the choice of medium and salt concentrations is critical (Isah et al., 2018). During *in vitro* culture of *Gloriosa superba*, higher concentrations of phosphates and calcium have resulted in the inhibition of alkaloid biosynthesis (Pandurangan and Philomina, 2010), which highlights the importance of the concentrations of media nutrients for achieving productivity in *in vitro* culture system. It has been observed in earlier studies that the sucrose concentration and osmoticum of the medium affect the physiological and metabolic activity of the cells and influence fresh weight and dry weight of the cells (Kim et al., 2001). The lower percentage growth with higher dry weight seen in our sucrose and inoculum size experiments could influence a combination of factors, including sucrose concentration, the osmolarity of the medium and the inoculum size.

Inoculum density is one of the important factors that could influence both biomass production and the productivity of the bioactive compound. This has been shown by Lee and Shuler (2000) while working with *Catharanthus roseus*. While working with *Stevia rebaudiana* Mathur and Shekhawat (2013) observed differences in cell biomass production at different inoculum densities. Our results also highlight that the inoculum density influences biomass production at the end of the growth period. The results (**Figure 7**) show that 3 g FW/100 mL of MS medium was the most suitable inoculum density for achieving the best biomass production.

Another critical condition for achieving optimum biomass generation in cell suspensions and at the same time, maintaining the productivity of the desired secondary metabolite, is that the growth medium is provided with the optimum ratio of auxin and cytokinin. The application of some of these plant growth regulators is limited by their classification as harmful to the environment and human health, especially the synthetic growth regulators, which are considered pesticides and their use is regulated by law around the world, such as in Europe (https://ec.europa.eu/food/plant/pesticides/eu-pesticides-database/public/?event=homepage&language=EN). Häkkinen et al. (2020) reported only trace amounts of free growth regulators are present in the cells at the harvesting point. At the same time, the relationship between auxin/cytokinin and the production of secondary metabolites has been highlighted in various studies (Baque et al., 2010; Kurepa et al., 2023). Auxins and cytokinins are closely linked with the production of reactive oxygen species (ROS) in cells which in turn is linked to secondary metabolite production (Huang et al., 2019). In feijoa cell suspensions, we observed that 2,4-D at 2 mg/L and 4 mg/L concentration, along with BAP at 0.2 mg/L, were the best concentrations for optimal growth (**Figure 8**). On the other hand, no growth of cell suspensions was observed in the absence of both the auxin and cytokinin, indicating that the growth of feijoa cell suspension depended on the availability of an exogenous supply of auxins and cytokinins. Further studies on the effect of 2,4-D and BAP on accumulating secondary metabolites in feijoa cell suspensions need to be investigated. Also, the possibilities of applying safer plant growth regulators such as coconut water and indole acetic acid or strategies that would minimise the application of harmful synthetic growth regulators to acceptable levels in combination with natural PGR should be investigated. Häkkinen et al. (2020) reported a two-phase culture system where the maintenance of cells could be with the original medium (which might include any synthetic plant growth regulators), whereas the scaling up of cells could be in plant growth regulator free medium.

Sugars act as energy sources and signal molecules that affect cultured cells’ growth, development, and metabolism (Rolland et al., 2006). Sugars are also classified as important regulatory molecules with signalling functions in plants. Earlier work (Wang and Weathers, 2007) has indicated that different plants have different abilities towards the uptake of different forms of sugars such as hexoses, glucose and fructose, and disaccharide sucrose, which can result in differential growth patterns even though in most cases of plant cell suspension cultures, sucrose is the most commonly used sugar. When cultured with fructose*, Phaseolus vulgaris* cell suspensions had a higher growth rate than glucose or sucrose (Botha and O’Kennedy, 1998). *Cynara cardunculus* cell suspension cultures could use glucose better than fructose (Oliviero et al., 2022). Cell wall invertase, cytoplasmic invertase and vacuolar invertase are the key enzymes regulating the breakdown of sucrose and its availability in downstream processes. These enzymes generate high levels of extracellular glucose and fructose that are taken up by hexose transporters (Rolland et al., 2006). The higher growth rate seen with glucose and fructose could be due to the higher affinity of the feijoa cells towards hexose transporters, as Rolland et al. (2006) suggested. Our experiments with different sucrose concentrations revealed that at 3% sucrose, the growth of feijoa cells was optimum. Further studies are required to understand carbohydrate assimilation, which would help optimise the medium for optimum biomass growth. Expression of secondary metabolites is intricately associated with their complex pathways. Numerous primary and secondary metabolites, such as products of the glyoxylate pathway, anthocyanin and artemisin, have responded differently to sugars (Arsenault et al., 2010). In other species, such as the Brazilian *Hancornia speciosa* (Gomes) cell suspension cultures, adding 3% glucose increased fresh weight and production of polyphenols (Dantas et al., 2021). The differences seen in the period for achieving the maximum growth during the growth curve between Amer et al. (2023) and the current study, 14 and 30 days, respectively, could be attributed to the differences in the varieties of feijoa, the different source tissue used for induction of callus as well as the differences in the set of growth conditions. Even though applications of feijoa cell suspensions for bioreactor setup have been mentioned recently (Amer et al., 2023) investigating the anticoagulant properties of feijoa, there has been a lack of data on the effect of different parameters involved in generating cell suspensions from feijoa. The current study has investigated and presented for the first time the effect of different parameters involved in establishing feijoa cell suspension cultures aimed at generating biomass for the extraction of bioactive compounds. The differences observed in the percentage growth rates under MS half strength, 3% sucrose and 2 mg/L of 2,4-D could be due to sucrose’s dual role, which serves as an energy source and an osmotic stabiliser. Sugars have been considered as signalling molecules that affect the growth, development, and metabolism of plant cell cultures (Murthy et al., 2014).

MeJA is considered a major secondary signalling molecule able to trigger signal transduction, leading to the generation of secondary signals that stimulate regulatory proteins, which coordinate the expression of biosynthetic genes (Zhao et al., 2005). Earlier studies have revealed that amongst the elicitors and concentrations, the use of 100 µM MeJA was found optimum for secondary metabolite enhancement in the majority of the experiments (Giri and Zaheer, 2016). The production of secondary metabolites typically occurs in the late stationary phase of the growth curve and is associated with growth inhibition and the production of enzymes for secondary metabolism (Ochoa-Villarreal et al., 2016). In our study, we evaluated 100 and 50 µM MeJA concentrations and the treatment of the feijoa cell suspension with 50 µM MeJA during the stationary phase after 30 days of growth, increasing the number of secondary metabolites and plant hormones. This increase in secondary metabolites could be due to multiple factors, as discussed by several authors.

In this study, we also reported an increased number of metabolites in cell cultures versus floral buds as compared to the previous work reported by Amer et al. (2023), in which they reported fewer total phenolic and flavonoids in the cell cultures from bioreactors when compared to the leaves. Interaction of signalling compounds such as jasmonic acid generates increased ROS, contributing to gene expression and the production of requisite transcripts to synthesise increased secondary metabolites (Giri and Zaheer, 2016). It is also known that chemical elicitors have the multitasking ability to drive a number of pathways at the biochemical and genetic levels (Boatwright and Pajerowska-Mukhtar, 2013). During the mechanism of elicitor action such as jasmonic acid, the key control points, which play a significant role, could be elicitor perception by the plasma membrane, a signal from organelles such as chloroplasts and mitochondria and participation of the 26S proteasomal degradation pathway facilitated by JA (Giri and Zaheer, 2016). Peng et al. (2022) identified 10 phenolic compounds in feijoa extracts, including phenolic acids, flavonoids and procyanidins, which play a significant role in the anticancer activity of the feijoa extract. Our study’s UPLC – QTOF MS analysis of floral buds and cell suspensions identified arctigenin, a high-value bioactive compound with pharmaceutical applications for treating various diseases. Arctigenin is a dibenzylbutyrolactone lignan, a phenolic compound and has been attributed with several therapeutic properties, including anti-tumor, anti-inflammatory, anti-colitis and anti-microbial effects (Shabgah et al., 2021). The identification of arctigenin in feijoa floral buds and cell cultures also compliments the strong anticancer activity of feijoa extract in earlier studies (Peng et al., 2022). Arctigenin has often been used as a lead compound for drug development because of its novel structure, pharmacological properties, and great developmental potential (Wu et al., 2022). Further evaluation with respect to the yield of arctigenin in the cell suspensions undergoing experiments with different types of elicitors and precursors, needs to be investigated.

## 5 Conclusion

We have identified the key parameters that could influence the establishment of feijoa cell suspension cultures from the various experiments conducted. Our experiments have identified the optimal salt concentrations in the medium, optimum inoculum density, the optimum 2,4-D concentrations in the medium and the best sugar sources and their concentrations for initiating and growing feijoa cell suspension cultures.

However, more studies are required to scope for alternative strategies with suitable plant growth regulators that would be readily acceptable for biomass generation for extracting high-value bioactive compounds at an industrial scale, considering the negative effects of 2,4-D on human health and the environment.

The phytochemical analysis of elicitor treated cell suspensions revealed increased phenolic compounds and secondary metabolites at 50µM MeJA. Our study’s identification and confirmation of arctigenin, a natural anticancer agent, also strongly supports exploring feijoa in *in vitro* cell suspension cultures for generating biomass to extract arctigenin and other high-value compounds. The presence of arctigenin supports previous studies in which anticancer properties in feijoa extracts have been identified. The presence of these types of high-value compounds in feijoa provides the foundation for the development of biomass production in bioreactors, which would lead to reduced environmental and ecological footprint and, at the same time, will allow harvest of these compounds year-round independently of biotic and abiotic factors and supply chain issues. Future studies will investigate establishing feijoa cell suspension cultures in bioreactors with acceptable plant growth regulators. Studies should also focus on identifying key elicitors which would further increase the production of arctigenin from the cells. More efforts should also be focused on revealing other pharmacological properties of the cell extracts derived from feijoa cell suspensions. The possibilities of extracting arctigenin from feijoa cell suspensions from bioreactors should also be explored.

## Data availability statement

The original contributions presented in the study are included in the article/**Supplementary Material**. Further inquiries can be directed to the corresponding author.

## Author contributions

SR: Conceptualization, Formal analysis, Investigation, Methodology, Project administration, Resources, Visualization, Writing – original draft, Writing – review & editing. II: Data curation, Formal analysis, Investigation, Methodology, Resources, Validation, Visualization, Writing – review & editing. SP: Data curation, Formal analysis, Investigation, Methodology, Validation, Writing – review & editing. HN: Data curation, Formal analysis, Investigation, Methodology, Validation, Writing – review & editing. SH: Funding acquisition, Writing – review & editing.
