# Supplementary material for: Establishment of feijoa (Acca sellowiana) callus and cell suspension cultures and identification of arctigenin - a high value bioactive compound
Source: Front Plant Sci. 2024 Jan 17;14:1281733. doi: 10.3389/fpls.2023.1281733 (PMC10829094; doi:10.3389/fpls.2023.1281733)
Supplement: Supplementary file 1 [file DataSheet_1.docx]

**SUPPLEMENTARY MATERIAL**

**Table S1. The gradient of the mobile phase of UPLC**

| **Time (min)** | **A (water + 0.1% FA) %** | **B (acetonitrile + 0.1% Formic Acid) %** |
| --- | --- | --- |
| 0 | 100 | 0 |
| 5 | 100 | 0 |
| 20 | 0 | 100 |

**Table S2. QTOF MS method settings**

| **Parameter** | **Value** |
| --- | --- |
| Gas Temperature (°C) | 350 |
| Gas Flow (L/min) | 9 |
| Nebulizer pressure (psig) | 35 |
| Sheath Gas Temperature (°C) | 320 |
| Sheath Gas Flow (L/min) | 9 |
| **ESI Source Parameters** |  |
| VCap | 3000 |
| Nozzle Voltage (V) | 2000 |
| Fragmentor | 85 |
| Skimmer1 | 45 |
| OctapoleRFPeak | 750 |

**Table S3A. Putatively identified compounds at 20 days of after MeJA elicitation (50 µM vs 100 µM vs ctrl), at different time points (0-72 hours).**

| **Methyl Jasmonate concentration (µM)** |  |  |  |  | **50** | | | | | | **100** | | | | | | **ctrl** | | | | | |
| --- | --- | --- | --- | --- | --- | --- | --- | --- | --- | --- | --- | --- | --- | --- | --- | --- | --- | --- | --- | --- | --- | --- |
| **Timeline (Hours)** |  |  |  |  | **0** | **6** | **12** | **24** | **48** | **72** | **0** | **6** | **12** | **24** | **48** | **72** | **0** | **6** | **12** | **24** | **48** | **72** |
| **Compound** | **Score** | **m/z** | **RT** | **Ion Specie** |  |  |  |  |  |  |  |  |  |  |  |  |  |  |  |  |  |  |
| (-)-Epicatechin gallate | 99.87 | 441.0806 | 9.17 | (M-H)- | √ | √ | √ | √ | √ | √ | √ | √ | √ | √ | √ | √ |  |  |  |  |  |  |
| (-)-Epigallocatechin gallate | 98.72 | 457.0762 | 8.75 | (M-H)- | √ | √ | √ | √ | √ | √ | √ | √ | √ | √ | √ | √ |  | √ | √ |  | √ | √ |
| (+)-Artemisinate | 99.94 | 279.1607 | 11.60 | (M+COOH)- | √ | √ | √ | √ | √ | √ | √ | √ | √ | √ | √ | √ | √ | √ | √ | √ | √ | √ |
| (+)-Secoisolariciresinol | 99.58 | 407.1175 | 10.10 | (M+COOH)- | √ | √ | √ | √ | √ | √ | √ | √ | √ | √ | √ | √ | √ | √ | √ | √ | √ | √ |
| (+)-Taxifolin | 99.19 | 303.0501 | 9.47 | (M-H)- | √ | √ | √ | √ | √ | √ | √ | √ | √ | √ | √ | √ | √ | √ | √ |  | √ | √ |
| (2R,3S,4S)-Leucocyanidin | 99.94 | 305.0670 | 7.85 | (M-H)- | √ | √ | √ | √ | √ | √ | √ | √ | √ | √ | √ | √ | √ | √ | √ | √ | √ | √ |
| (2S,4As,6aS,6bR,10S,12aS)-10-hydroxy-2-(hydroxymethyl)-2,4a,6a,6b,9,9,12a-heptamethyl-3,4,5,6,6a,7,8,8a,10,11,12,14b-dodecahydro-1H-picen-13-one | 99.94 | 455.3523 | 14.73 | (M-H)- | √ | √ | √ | √ | √ | √ | √ | √ | √ | √ | √ | √ | √ | √ | √ | √ | √ | √ |
| (E)-6'-Hydroxyferulate | 98.24 | 209.0449 | 8.14 | (M-H)- | √ | √ | √ | √ | √ | √ | √ | √ | √ | √ | √ | √ |  |  |  |  |  |  |
| (S)-laudanosine | 99.94 | 356.1861 | 11.27 | (M-H)- |  |  |  | √ | √ | √ |  |  | √ | √ | √ | √ |  |  |  |  |  |  |
| 1,2,6-Trigalloylglucose | 95.91 | 635.0881 | 8.64 | (M-H)- | √ | √ | √ | √ | √ | √ | √ | √ | √ | √ | √ | √ |  |  |  |  |  | √ |
| 1,2-Di-O-sinapoyl-beta-D-glucose | 98.82 | 591.1685 | 9.89 | (M-H)- |  | √ | √ | √ | √ | √ |  | √ | √ | √ |  |  |  |  |  |  |  |  |
| 1,6-Bis-O-galloyl-beta-D-glucose | 99.80 | 483.0775 | 8.29 | (M-H)- | √ | √ | √ | √ | √ | √ | √ | √ | √ | √ | √ | √ |  |  |  |  |  |  |
| 16-alpha-Hydroxygypsogenate | 92.44 | 501.3211 | 11.30 | (M+COOH)- | √ | √ | √ | √ | √ | √ | √ | √ | √ | √ | √ | √ | √ | √ | √ | √ | √ | √ |
| 1-O-Vanilloyl-beta-D-glucose | 99.87 | 329.0868 | 8.14 | (M-H)- | √ | √ | √ | √ | √ | √ | √ | √ | √ | √ | √ | √ | √ | √ | √ |  | √ |  |
| 2-Coumarate | 99.77 | 163.0395 | 8.99 | (M-H)- | √ | √ | √ | √ | √ | √ | √ | √ | √ | √ | √ | √ | √ | √ | √ |  | √ | √ |
| 2'-Hydroxy 3,6,7,3',4'-pentamethylquercetagetin | 99.71 | 449.1075 | 9.25 | (M+COOH)- | √ | √ | √ | √ | √ | √ | √ | √ | √ | √ | √ | √ | √ | √ | √ | √ | √ | √ |
| 2-Hydroxy-2,3-dihydrogenistein-7-olate | 99.81 | 287.0555 | 9.92 | (M-H)- | √ | √ | √ | √ | √ | √ | √ | √ | √ | √ | √ | √ | √ | √ | √ | √ | √ | √ |
| 2-Methoxycarbonylphenyl b-D-glucopyranoside | 99.91 | 359.0973 | 8.17 | (M+COOH)- | √ | √ | √ | √ | √ | √ | √ | √ | √ | √ | √ | √ | √ | √ |  |  |  |  |
| 2-Phenylethyl beta-primeveroside | 88.35 | 415.1605 | 9.28 | (M-H)- |  |  |  | √ |  |  |  |  | √ | √ | √ |  |  |  |  |  |  |  |
| 3,5-Di-C-glucosyl-2,4,4',6-tetrahydroxydibenzoylmethane | 98.29 | 611.1569 | 8.88 | (M-H)- | √ | √ | √ | √ | √ | √ | √ | √ | √ | √ | √ | √ | √ | √ |  |  | √ |  |
| 3',5'-Di-C-glucosylphloretin | 99.76 | 643.1876 | 7.92 | (M+COOH)- | √ | √ | √ | √ | √ | √ | √ | √ | √ | √ | √ | √ | √ | √ |  |  |  |  |
| 3,5-Didehydroshikimate | 99.40 | 169.0137 | 4.48 | (M-H)- | √ | √ | √ | √ | √ | √ | √ | √ |  |  |  |  |  |  |  |  |  |  |
| 3,6,7,2',4'-Pentamethylquercetagetin 3'-O-beta-D-glucoside | 97.18 | 611.1577 | 8.88 | (M+COOH)- | √ | √ | √ | √ | √ | √ | √ | √ | √ | √ | √ | √ | √ | √ |  |  | √ |  |
| 3-beta-Hydroxyglycyrrhetinate | 99.93 | 515.3365 | 12.57 | (M+COOH)- | √ | √ | √ | √ | √ | √ | √ | √ | √ | √ | √ | √ | √ | √ | √ | √ | √ | √ |
| 3-Hydroxy-5-methoxybiphenyl | 99.87 | 245.0813 | 8.43 | (M+COOH)- | √ | √ | √ | √ | √ | √ | √ | √ | √ | √ | √ | √ | √ | √ | √ |  | √ |  |
| 3-Hydroxy-beta-ionone | 99.38 | 253.1439 | 11.29 | (M+COOH)- | √ | √ | √ | √ | √ | √ | √ | √ | √ | √ | √ | √ | √ | √ | √ | √ | √ | √ |
| 3-Isobutanoyl-3',4-di(isovaleryl)sucrose | 97.90 | 579.2644 | 8.90 | (M+COOH)- |  |  |  | √ | √ | √ |  |  |  | √ | √ | √ |  |  |  |  |  |  |
| 3-Lauryl-3'-isobutanoyl-4-(isovaleryl)sucrose | 99.36 | 723.3783 | 11.96 | (M+COOH)- | √ | √ | √ | √ | √ | √ | √ | √ | √ | √ | √ | √ |  |  |  |  |  |  |
| 3''-O-(4-Hydroxycinnamoyl)astragalin | 99.93 | 593.1282 | 8.20 | (M-H)- | √ | √ | √ | √ | √ | √ | √ | √ | √ | √ | √ | √ | √ | √ | √ |  | √ |  |
| 4-(Isobutanoyl)sucrose | 97.31 | 411.1492 | 8.44 | (M-H)- |  |  |  | √ | √ | √ |  |  |  | √ | √ | √ |  |  |  |  |  |  |
| 4alpha-Carboxy-5alpha-cholesta-7,24-dien-3beta-ol | 99.90 | 473.327 | 11.77 | (M+COOH)- | √ | √ | √ | √ | √ | √ | √ | √ | √ | √ | √ | √ | √ | √ | √ | √ | √ | √ |
| 4alpha-Carboxy-ergosta-7,24(241)-dien-3beta-ol | 99.90 | 487.3419 | 11.77 | (M+COOH)- | √ | √ | √ | √ | √ | √ | √ | √ | √ | √ | √ | √ | √ | √ | √ | √ | √ | √ |
| 4alpha-Formyl-ergosta-7,24(241)-dien-3beta-ol | 99.97 | 471.3469 | 13.30 | (M+COOH)- | √ | √ | √ | √ | √ | √ | √ | √ | √ | √ | √ | √ | √ | √ | √ | √ | √ | √ |
| 4alpha-Hydroxymethyl-4beta-methyl-5alpha-cholesta-8,24-dien-3beta-ol | 99.35 | 473.3623 | 12.62 | (M+COOH)- | √ | √ | √ | √ | √ | √ | √ | √ | √ | √ | √ | √ | √ | √ | √ |  | √ | √ |
| 4'-Demethylepipodophyllotoxin | 98.24 | 445.1131 | 8.84 | (M+COOH)- | √ | √ |  | √ | √ | √ |  | √ | √ | √ | √ | √ |  |  |  |  |  |  |
| 6-O-Galloyl-beta-D-glucose | 98.75 | 331.0661 | 4.48 | (M-H)- | √ | √ | √ | √ | √ | √ | √ | √ | √ | √ |  |  |  |  |  |  |  | √ |
| 9'-Hoaba | 89.24 | 279.121 | 8.89 | (M-H)- |  |  |  | √ | √ | √ |  |  |  | √ | √ | √ |  |  |  |  |  |  |
| Abscisic alcohol | 99.93 | 249.1488 | 13.85 | (M-H)- | √ | √ | √ | √ | √ | √ | √ | √ | √ | √ | √ | √ | √ | √ | √ | √ | √ | √ |
| Alizarin | 90.40 | 285.0401 | 9.35 | (M+COOH)- | √ | √ | √ | √ | √ | √ | √ | √ | √ | √ | √ | √ | √ | √ |  |  |  |  |
| alpha-3',4'-Anhydrovinblastine | 96.20 | 791.3994 | 10.22 | (M-H)- | √ | √ | √ | √ | √ | √ | √ | √ | √ | √ | √ | √ |  |  |  |  |  |  |
| alpha-3',4'-Anhydrovinblastine radical | 85.69 | 789.3888 | 10.57 | (M-H)- |  |  |  | √ | √ | √ |  |  |  | √ | √ | √ |  |  |  |  |  |  |
| Arctigenin | 100.00 | 371.1512 | 15.24 | (M-H)- | √ | √ | √ | √ | √ | √ | √ | √ | √ | √ | √ | √ | √ | √ | √ | √ | √ | √ |
| Artemisinin | 97.21 | 281.1391 | 9.48 | (M-H)- |  |  |  | √ |  |  |  |  |  | √ |  |  |  |  |  |  |  |  |
| Astilbin (Taxifolin 3-O-rhamnoside) | 99.71 | 449.1075 | 9.27 | (M-H)- | √ | √ | √ | √ | √ | √ | √ | √ | √ | √ | √ | √ | √ | √ | √ | √ | √ | √ |
| Avenastenone | 99.94 | 455.3523 | 14.73 | (M+COOH)- | √ | √ | √ | √ | √ | √ | √ | √ | √ | √ | √ | √ | √ | √ | √ | √ | √ | √ |
| Baicalin | 99.44 | 491.0821 | 8.90 | (M+COOH)- | √ | √ | √ | √ | √ | √ | √ | √ | √ | √ | √ | √ |  |  |  |  |  |  |
| Benzoyl-beta-D-glucoside | 99.87 | 329.0868 | 8.14 | (M+COOH)- | √ | √ | √ | √ | √ | √ | √ | √ | √ | √ | √ | √ | √ | √ | √ |  | √ |  |
| Biliverdine | 86.60 | 581.2372 | 9.10 | (M-H)- |  | √ | √ | √ | √ | √ |  | √ | √ | √ | √ | √ |  |  |  |  |  |  |
| Catechin | 99.75 | 289.0711 | 8.50 | (M-H)- | √ | √ | √ | √ | √ | √ | √ | √ | √ | √ | √ | √ | √ | √ | √ |  |  |  |
| Chalconaringenin 4'-glucoside | 97.12 | 433.1125 | 9.62 | (M-H)- | √ | √ | √ | √ | √ | √ | √ | √ | √ | √ | √ | √ | √ | √ |  | √ |  |  |
| cis-Zeatin | 93.36 | 218.1026 | 7.65 | (M-H)- | √ |  |  | √ | √ | √ |  | √ |  | √ | √ | √ |  |  |  |  |  |  |
| cis-zeatin-7-N-glucoside | 93.43 | 380.1588 | 14.68 | (M-H)- |  | √ | √ | √ | √ | √ | √ | √ | √ | √ | √ |  | √ | √ | √ |  | √ | √ |
| Combretol | 97.12 | 433.1125 | 9.62 | (M+COOH)- | √ | √ | √ | √ | √ | √ | √ | √ | √ | √ | √ | √ | √ | √ | √ | √ | √ | √ |
| Cyanidin 3-O-(6'-O-malyl-beta-D-glucoside) | 93.81 | 609.1142 | 9.54 | (M-H)- |  |  |  |  |  |  |  |  |  |  |  |  | √ | √ |  |  |  |  |
| Cyanidin 3-O-beta-D-caffeoylglucoside | 99.18 | 609.1226 | 7.84 | (M-H)- | √ | √ | √ | √ | √ | √ | √ | √ |  |  |  | √ | √ | √ |  |  |  |  |
| Daphnetin | 99.90 | 177.0188 | 9.60 | (M-H)- | √ | √ | √ | √ | √ | √ | √ | √ | √ | √ | √ | √ | √ | √ | √ | √ | √ | √ |
| Dicrocin | 99.29 | 651.2668 | 9.44 | (M+COOH)- |  | √ | √ | √ | √ | √ |  | √ | √ | √ | √ | √ |  |  |  |  |  |  |
| Dihydroprecondylocarpine acetate | 94.12 | 441.202 | 15.39 | (M-H)- | √ | √ | √ | √ | √ | √ | √ | √ | √ | √ | √ | √ | √ | √ | √ | √ | √ | √ |
| Diphyllin | 99.10 | 425.0863 | 8.02 | (M+COOH)- | √ | √ |  | √ | √ | √ | √ | √ |  |  | √ |  | √ | √ |  |  |  |  |
| Eugeniin | 97.34 | 937.0951 | 8.89 | (M-H)- | √ | √ |  |  |  |  |  |  |  |  |  |  |  |  |  |  |  |  |
| Eugenyl-GXG | 94.67 | 665.2251 | 8.58 | (M+COOH)- |  |  |  |  | √ | √ |  |  |  | √ | √ | √ |  |  |  |  |  |  |
| Gaultherin | 99.90 | 491.1391 | 8.02 | (M-H)- | √ | √ | √ | √ | √ | √ | √ | √ | √ | √ | √ | √ | √ | √ | √ |  | √ |  |
| Gentisate 5-O-beta-D-xylopyranoside | 98.79 | 331.0659 | 4.48 | (M+COOH)- | √ | √ | √ | √ | √ | √ | √ | √ | √ | √ |  |  |  |  |  |  |  | √ |
| Geranyl 6-O-beta-D-xylopyranosyl-beta-D-glucopyranoside | 99.62 | 447.2233 | 10.70 | (M-H)- |  |  |  | √ |  |  |  |  | √ | √ | √ | √ |  |  |  |  |  |  |
| Gingerol | 99.89 | 293.175 | 11.57 | (M-H)- | √ | √ | √ | √ | √ | √ | √ | √ | √ | √ | √ | √ | √ | √ | √ | √ | √ | √ |
| Glycyrrhetinic aldehyde | 99.53 | 453.3368 | 14.32 | (M+H)- | √ | √ | √ | √ | √ | √ | √ | √ | √ | √ | √ | √ | √ | √ | √ | √ | √ | √ |
| Gypsogenate | 99.38 | 531.3326 | 12.12 | (M+COOH)- | √ | √ | √ | √ | √ | √ | √ | √ | √ | √ | √ | √ | √ | √ | √ | √ | √ | √ |
| Hederagenin | 99.97 | 471.3469 | 13.30 | (M-H)- | √ | √ | √ | √ | √ | √ | √ | √ | √ | √ | √ | √ | √ | √ | √ | √ | √ | √ |
| Hispidol-4'-O-beta-D-glucoside | 97.13 | 461.1098 | 8.40 | (M+COOH)- | √ |  |  |  |  |  |  |  |  |  |  |  |  |  |  |  |  |  |
| Jasmonate | 99.75 | 209.1167 | 10.93 | (M-H)- | √ | √ | √ | √ | √ | √ | √ | √ | √ | √ | √ | √ |  |  |  |  |  |  |
| Linamarin | 99.28 | 246.0975 | 8.09 | (M-H)- | √ | √ |  | √ | √ | √ |  | √ | √ | √ | √ | √ | √ |  |  |  |  |  |
| Macarpine | 97.17 | 436.1042 | 7.96 | (M+COOH)- | √ | √ | √ | √ | √ | √ | √ | √ | √ | √ | √ | √ | √ | √ | √ | √ | √ | √ |
| Mermesin | 99.87 | 245.0813 | 8.43 | (M-H)- | √ | √ | √ | √ | √ | √ | √ | √ | √ | √ | √ | √ | √ | √ | √ |  | √ | √ |
| Methylsalicyl-GXG | 95.97 | 653.1913 | 7.55 | (M+COOH)- |  |  |  |  |  |  |  | √ |  |  |  |  |  |  |  |  |  |  |
| Naringenin chalcone | 99.86 | 271.0607 | 10.63 | (M-H)- | √ | √ | √ | √ | √ | √ | √ | √ | √ | √ | √ | √ | √ | √ | √ | √ | √ | √ |
| Neolinustatin | 97.12 | 468.1711 | 8.10 | (M+COOH)- |  | √ | √ | √ | √ | √ |  |  | √ | √ | √ | √ |  |  |  |  |  |  |
| Nothofagin | 99.94 | 435.1284 | 9.21 | (M-H)- | √ | √ | √ | √ | √ | √ |  | √ | √ | √ | √ | √ | √ | √ |  |  |  |  |
| Olivetol | 99.81 | 225.113 | 9.34 | (M+COOH)- |  | √ | √ | √ | √ | √ |  | √ | √ | √ | √ | √ |  |  |  |  |  |  |
| Pentagalloylglucose | 98.50 | 939.11 | 9.17 | (M-H)- | √ | √ | √ |  |  |  | √ | √ |  |  |  |  |  |  |  |  |  |  |
| Peonidin-3-glucoside | 93.16 | 461.1098 | 8.40 | (M-H)- | √ |  |  |  |  |  |  |  |  |  |  |  |  |  |  |  |  |  |
| Phloretin | 99.07 | 273.0754 | 8.91 | (M-H)- | √ |  |  |  |  |  |  | √ |  |  |  |  |  |  |  |  |  |  |
| Pinostrobin | 99.78 | 315.0864 | 9.21 | (M+COOH)- | √ | √ |  | √ | √ | √ | √ | √ | √ | √ | √ | √ |  |  |  |  |  |  |
| Procyanidin B2 | 99.92 | 577.1338 | 8.22 | (M-H)- | √ | √ | √ | √ | √ | √ | √ | √ | √ | √ | √ | √ | √ | √ | √ |  | √ | √ |
| Quercetagetin 7-O-glucoside | 99.72 | 479.0823 | 7.87 | (M-H)- |  |  |  |  |  |  |  | √ |  |  |  |  |  |  |  |  |  |  |
| Quercetin | 86.58 | 301.0346 | 9.55 | (M-H)- |  |  |  |  |  |  | √ |  |  |  |  |  |  |  |  |  |  |  |
| Rishitin | 99.91 | 221.1539 | 11.57 | (M-H)- | √ | √ | √ | √ | √ | √ | √ | √ | √ | √ | √ | √ | √ | √ | √ | √ | √ | √ |
| salicyl-6-hydroxy-2-cyclohexene-on-oyl | 98.58 | 261.0757 | 7.87 | (M-H)- | √ | √ | √ | √ | √ | √ | √ | √ | √ | √ | √ | √ | √ | √ |  |  |  |  |
| Sissotrin | 99.09 | 445.1131 | 8.84 | (M-H)- | √ | √ | √ | √ | √ | √ |  | √ | √ | √ | √ | √ |  |  |  |  |  |  |
| Tetraketide pyrone | 99.07 | 223.0975 | 8.16 | (M-H)- |  |  |  |  | √ | √ |  |  |  |  | √ | √ |  |  |  |  |  |  |
| trans-Tuberonic acid glucoside | 97.97 | 387.1653 | 8.41 | (M-H)- |  | √ | √ | √ | √ | √ |  | √ | √ | √ | √ | √ |  |  |  |  |  |  |
| Tuberonate | 99.81 | 225.113 | 9.34 | (M-H)- |  | √ | √ | √ | √ | √ |  | √ | √ | √ | √ | √ |  |  |  |  |  |  |
| Vernolic acid | 99.05 | 341.2331 | 11.01 | (M+COOH)- |  |  |  |  |  | √ |  |  |  |  |  |  |  |  |  |  |  |  |
| Vincristine | 98.79 | 823.3891 | 9.60 | (M-H)- | √ | √ |  | √ |  |  | √ | √ |  |  |  |  |  |  |  |  |  |  |

**Table S3B. Putatively identified compounds at 25 days after MeJA elicitation (50 µM vs 100 µM vs ctrl), at different time points (0-72 hours).**

| **Methyl Jasmonate concentration (µM)** |  |  |  |  | **50** | | | | | | **100** | | | | | | **ctrl** | | | | | |
| --- | --- | --- | --- | --- | --- | --- | --- | --- | --- | --- | --- | --- | --- | --- | --- | --- | --- | --- | --- | --- | --- | --- |
| **Timeline (Hours)** |  |  |  |  | **0** | **6** | **12** | **24** | **48** | **72** | **0** | **6** | **12** | **24** | **48** | **72** | **0** | **6** | **12** | **24** | **48** | **72** |
| **Compound** | **Score** | **m/z** | **RT** | **Ion Specie** |  |  |  |  |  |  |  |  |  |  |  |  |  |  |  |  |  |  |
| (-)-Epicatechin gallate | 99.87 | 441.0806 | 9.17 | (M-H)- | √ | √ | √ | √ | √ | √ | √ | √ | √ | √ | √ | √ | √ | √ | √ | √ | √ | √ |
| (-)-Epigallocatechin gallate | 98.72 | 457.0762 | 8.75 | (M-H)- | √ | √ | √ | √ | √ | √ | √ | √ | √ | √ | √ |  | √ | √ | √ | √ | √ | √ |
| (+)-Artemisinate | 99.94 | 279.1607 | 11.60 | (M+COOH)- | √ | √ | √ | √ | √ | √ | √ | √ | √ | √ | √ | √ | √ | √ | √ | √ | √ | √ |
| (+)-Secoisolariciresinol | 99.58 | 407.1175 | 10.10 | (M+COOH)- |  | √ |  |  |  | √ |  |  |  | √ |  | √ | √ | √ | √ | √ | √ | √ |
| (+)-Taxifolin | 99.19 | 303.0501 | 9.470 | (M-H)- | √ | √ | √ | √ | √ | √ | √ | √ | √ | √ | √ | √ | √ | √ | √ | √ | √ | √ |
| (2S,4As,6aS,6bR,10S,12aS)-10-hydroxy-2-(hydroxymethyl)-2,4a,6a,6b,9,9,12a-heptamethyl-3,4,5,6,6a,7,8,8a,10,11,12,14b-dodecahydro-1H-picen-13-one | 99.94 | 455.3523 | 14.73 | (M-H)- | √ | √ | √ | √ | √ | √ | √ | √ | √ | √ | √ | √ | √ | √ | √ | √ | √ | √ |
| (E)-6'-Hydroxyferulate | 98.24 | 209.0449 | 8.14 | (M-H)- | √ | √ | √ | √ | √ |  | √ | √ | √ | √ | √ | √ | √ | √ | √ | √ | √ | √ |
| (S)-laudanosine | 99.94 | 356.1861 | 11.27 | (M-H)- |  |  | √ | √ | √ | √ |  |  | √ | √ | √ | √ |  |  |  |  |  |  |
| 1,2,6-Trigalloylglucose | 95.91 | 635.0881 | 8.64 | (M-H)- | √ | √ | √ | √ |  |  | √ | √ |  |  |  |  | √ | √ | √ | √ | √ | √ |
| 1,6-Bis-O-galloyl-beta-D-glucose | 98.82 | 483.0771 | 8.22 | (M-H)- | √ | √ | √ | √ | √ | √ | √ | √ | √ |  |  |  | √ | √ | √ | √ | √ | √ |
| 16-alpha-Hydroxygypsogenate | 92.44 | 501.3211 | 11.30 | (M+COOH)- | √ | √ | √ | √ | √ | √ | √ | √ | √ | √ | √ | √ | √ | √ | √ | √ | √ | √ |
| 1-O-Vanilloyl-beta-D-glucose | 99.87 | 329.0868 | 8.14 | (M-H)- | √ | √ | √ | √ | √ | √ | √ | √ | √ | √ | √ | √ | √ | √ | √ | √ | √ | √ |
| 2-Coumarate | 99.77 | 163.0395 | 8.99 | (M-H)- |  | √ | √ | √ | √ | √ | √ |  | √ | √ |  | √ | √ | √ | √ | √ | √ | √ |
| 2'-Hydroxy 3,6,7,3',4'-pentamethylquercetagetin | 99.71 | 449.1075 | 9.25 | (M+COOH)- | √ | √ | √ | √ | √ | √ | √ | √ | √ |  |  |  | √ | √ | √ | √ | √ | √ |
| 2-Hydroxy-2,3-dihydrogenistein-7-olate | 99.81 | 287.0555 | 9.92 | (M-H)- | √ | √ | √ | √ | √ | √ | √ | √ | √ | √ | √ | √ | √ | √ | √ | √ | √ | √ |
| 2-Methoxycarbonylphenyl b-D-glucopyranoside | 99.91 | 359.0973 | 8.17 | (M+COOH)- | √ | √ | √ | √ | √ | √ | √ | √ | √ | √ | √ | √ | √ | √ | √ | √ | √ | √ |
| 3,5-Di-C-glucosyl-2,4,4',6-tetrahydroxydibenzoylmethane | 98.29 | 611.1569 | 8.88 | (M-H)- | √ | √ | √ | √ | √ | √ |  |  |  |  |  |  |  |  |  |  |  |  |
| 3',5'-Di-C-glucosylphloretin | 99.76 | 643.1876 | 7.92 | (M+COOH)- | √ | √ | √ | √ | √ | √ | √ | √ | √ | √ | √ | √ | √ | √ | √ | √ | √ | √ |
| 3,5-Didehydroshikimate | 99.40 | 169.0137 | 4.48 | (M-H)- |  | √ | √ |  |  |  | √ |  |  |  |  |  |  |  | √ |  |  | √ |
| 3,6,7,2',4'-Pentamethylquercetagetin 3'-O-beta-D-glucoside | 97.18 | 611.1577 | 8.88 | (M+COOH)- | √ | √ | √ | √ | √ | √ |  |  |  |  |  |  |  |  |  |  |  |  |
| 3-beta-Hydroxyglycyrrhetinate | 99.93 | 515.3365 | 12.57 | (M+COOH)- | √ | √ | √ | √ | √ | √ | √ | √ | √ | √ | √ | √ | √ | √ | √ | √ | √ | √ |
| 3-Hydroxy-5-methoxybiphenyl | 99.87 | 245.0813 | 8.43 | (M+COOH)- | √ | √ | √ | √ | √ | √ | √ | √ | √ | √ | √ |  | √ | √ | √ | √ | √ | √ |
| 3-Hydroxy-beta-ionone | 99.38 | 253.1439 | 11.29 | (M+COOH)- | √ | √ | √ | √ | √ | √ | √ | √ | √ | √ | √ | √ | √ | √ | √ | √ | √ | √ |
| 3-Isobutanoyl-3',4-di(isovaleryl)sucrose | 97.90 | 579.2644 | 8.90 | (M+COOH)- |  |  |  | √ | √ | √ |  |  |  | √ | √ | √ |  |  |  |  |  |  |
| 3-Lauryl-3'-isobutanoyl-4-(isovaleryl)sucrose | 99.36 | 723.3783 | 11.96 | (M+COOH)- | √ | √ | √ | √ | √ | √ | √ | √ | √ | √ | √ | √ | √ | √ | √ | √ | √ | √ |
| 3''-O-(4-Hydroxycinnamoyl)astragalin | 99.93 | 593.1282 | 8.20 | (M-H)- | √ | √ | √ | √ | √ | √ | √ | √ | √ | √ | √ | √ | √ | √ | √ | √ | √ | √ |
| 4-(Isobutanoyl)sucrose | 97.31 | 411.1492 | 8.44 | (M-H)- | √ | √ |  | √ |  | √ | √ |  |  | √ | √ | √ |  |  |  | √ |  |  |
| 4alpha-Carboxy-5alpha-cholesta-7,24-dien-3beta-ol | 99.90 | 473.327 | 11.77 | (M+COOH)- | √ | √ | √ | √ | √ | √ | √ | √ | √ | √ | √ | √ | √ | √ | √ | √ | √ | √ |
| 4alpha-Carboxy-ergosta-7,24(241)-dien-3beta-ol | 99.90 | 487.3419 | 11.77 | (M+COOH)- | √ | √ | √ | √ | √ | √ | √ | √ | √ | √ | √ | √ | √ | √ | √ | √ | √ | √ |
| 4alpha-Formyl-ergosta-7,24(241)-dien-3beta-ol | 99.97 | 471.3469 | 13.30 | (M+COOH)- | √ | √ | √ | √ | √ | √ | √ | √ | √ | √ | √ | √ | √ | √ | √ | √ | √ | √ |
| 4alpha-Hydroxymethyl-4beta-methyl-5alpha-cholesta-8,24-dien-3beta-ol | 99.35 | 473.3623 | 12.62 | (M+COOH)- | √ | √ | √ | √ | √ | √ | √ | √ | √ | √ | √ | √ |  |  |  |  |  |  |
| 4-Coumaroylshikimate | 99.07 | 319.0813 | 8.60 | (M-H)- | √ | √ | √ | √ | √ | √ |  |  |  |  |  |  | √ |  | √ | √ |  |  |
| 4'-Demethylepipodophyllotoxin | 98.24 | 445.1131 | 8.84 | (M+COOH)- |  |  |  |  |  |  | √ | √ | √ |  |  |  |  |  |  |  |  |  |
| 5-O-Caffeoylshikimate | 98.87 | 335.0763 | 8.46 | (M-H)- | √ | √ |  |  |  | √ |  |  |  |  |  |  | √ | √ | √ | √ | √ | √ |
| 6-O-Galloyl-beta-D-glucose | 98.75 | 331.0661 | 4.48 | (M-H)- | √ |  |  |  |  |  | √ | √ | √ | √ |  |  |  |  |  |  |  |  |
| 9,10,18-Trihydroxystearate | 93.48 | 331.2479 | 11.40 | (M-H)- |  |  |  |  |  |  |  |  |  |  |  |  |  |  |  | √ |  |  |
| 9'-Hoaba | 89.24 | 279.121 | 8.90 | (M-H)- |  | √ |  | √ | √ | √ |  | √ |  | √ | √ | √ |  |  |  |  |  |  |
| Abscisic alcohol | 99.93 | 249.1488 | 13.85 | (M-H)- | √ | √ | √ | √ | √ | √ | √ | √ | √ | √ | √ | √ | √ | √ | √ | √ | √ | √ |
| Alizarin | 90.40 | 285.0401 | 9.35 | (M+COOH)- | √ | √ | √ | √ | √ | √ | √ |  | √ | √ | √ |  | √ | √ | √ | √ |  | √ |
| alpha-3',4'-Anhydrovinblastine | 96.20 | 791.3994 | 10.22 | (M-H)- | √ | √ | √ | √ | √ | √ |  |  |  |  |  |  |  |  |  |  |  |  |
| alpha-3',4'-Anhydrovinblastine radical | 85.69 | 789.3888 | 10.57 | (M-H)- |  |  |  | √ | √ | √ | √ |  | √ | √ |  | √ |  |  |  |  | √ |  |
| Arctigenin | 100.00 | 371.1512 | 15.24 | (M-H)- | √ | √ | √ | √ | √ | √ | √ | √ | √ | √ | √ | √ | √ | √ | √ | √ | √ | √ |
| Artemisinin | 97.21 | 281.1391 | 9.48 | (M-H)- |  |  |  |  |  |  |  |  | √ |  |  |  |  |  |  |  |  |  |
| Astilbin (Taxifolin 3-O-rhamnoside) | 99.71 | 449.1075 | 9.270 | (M-H)- | √ | √ | √ | √ | √ | √ | √ | √ | √ |  |  |  | √ | √ | √ | √ | √ | √ |
| Avenastenone | 99.94 | 455.3523 | 14.73 | (M+COOH)- | √ | √ | √ | √ | √ | √ | √ | √ | √ | √ | √ | √ | √ | √ | √ | √ | √ | √ |
| Baicalin | 99.44 | 491.0821 | 8.90 | (M+COOH)- | √ | √ | √ | √ | √ | √ |  |  |  |  |  |  |  |  |  |  |  |  |
| Benzoyl-beta-D-glucoside | 99.87 | 329.0868 | 8.14 | (M+COOH)- | √ | √ | √ | √ | √ | √ | √ | √ | √ | √ | √ | √ | √ | √ | √ | √ | √ | √ |
| Bergapten | 84.80 | 215.0341 | 8.72 | (M+COOH)- |  |  | √ |  |  |  |  |  |  |  |  |  |  |  |  |  |  |  |
| Biliverdine | 86.60 | 581.2372 | 9.095 | (M-H)- |  | √ | √ | √ | √ | √ |  |  | √ | √ | √ | √ |  |  |  |  |  |  |
| C19-Gibberellin skeleton | 80.29 | 361.1627 | 11.56 | (M+COOH)- |  |  |  |  |  |  |  |  |  |  |  |  | √ |  |  |  |  |  |
| Catechin | 99.75 | 289.0711 | 8.50 | (M-H)- | √ | √ | √ | √ | √ | √ | √ | √ | √ | √ | √ | √ | √ | √ | √ | √ | √ | √ |
| Chalconaringenin 4'-glucoside | 97.12 | 433.1125 | 9.62 | (M-H)- | √ | √ | √ | √ | √ | √ |  |  |  |  |  |  | √ | √ | √ | √ | √ | √ |
| cis-Zeatin | 93.36 | 218.1026 | 7.65 | (M-H)- | √ |  | √ | √ | √ | √ |  |  |  |  | √ |  |  |  |  |  |  |  |
| cis-zeatin-7-N-glucoside | 93.43 | 380.1588 | 14.68 | (M-H)- | √ | √ | √ | √ | √ | √ | √ | √ | √ | √ | √ | √ | √ | √ | √ | √ | √ | √ |
| Combretol | 97.12 | 433.1125 | 9.62 | (M+COOH)- | √ | √ | √ | √ | √ | √ |  |  |  |  |  |  | √ | √ | √ | √ | √ | √ |
| Coproporphyrinogen III | 94.56 | 659.3132 | 14.72 | (M-H)- |  | √ |  | √ | √ |  |  |  |  | √ | √ | √ | √ | √ | √ | √ | √ | √ |
| Cyanidin 3-O-(6'-O-malyl-beta-D-glucoside) | 93.81 | 609.1142 | 9.54 | (M-H)- | √ | √ |  | √ | √ | √ |  |  |  | √ |  | √ |  |  |  |  |  |  |
| Cyanidin 3-O-beta-D-caffeoylglucoside | 99.18 | 609.1226 | 7.84 | (M-H)- | √ | √ | √ | √ | √ | √ | √ | √ | √ | √ | √ | √ | √ | √ | √ | √ |  | √ |
| Daphnetin | 99.90 | 177.0188 | 9.60 | (M-H)- | √ | √ | √ | √ | √ | √ | √ |  | √ |  | √ |  |  |  | √ | √ | √ |  |
| Dhurrin | 97.10 | 310.09 | 9.39 | (M-H)- |  |  |  |  |  |  |  |  | √ | √ | √ | √ |  |  |  |  |  |  |
| Dicrocin | 99.29 | 651.2668 | 9.44 | (M+COOH)- |  | √ | √ | √ | √ | √ |  | √ | √ | √ | √ | √ |  | √ | √ | √ | √ |  |
| Dihydroprecondylocarpine acetate | 94.12 | 441.202 | 15.39 | (M-H)- | √ | √ | √ | √ | √ | √ | √ | √ | √ | √ | √ | √ | √ | √ | √ | √ | √ | √ |
| Diphyllin | 99.10 | 425.0863 | 8.02 | (M+COOH)- | √ | √ | √ | √ | √ | √ | √ |  | √ |  |  |  | √ | √ | √ | √ | √ | √ |
| Eugenyl-GXG | 94.67 | 665.2251 | 8.58 | (M+COOH)- |  |  |  |  |  |  |  |  |  |  | √ | √ |  |  |  |  |  |  |
| Gaultherin | 99.90 | 491.1391 | 8.02 | (M-H)- | √ | √ | √ | √ | √ | √ | √ | √ | √ | √ | √ | √ | √ | √ | √ | √ | √ | √ |
| Gentisate 5-O-beta-D-xylopyranoside | 98.79 | 331.0659 | 4.48 | (M+COOH)- | √ |  |  |  |  |  | √ | √ | √ |  |  |  |  |  |  | √ |  |  |
| Geranyl 6-O-beta-D-xylopyranosyl-beta-D-glucopyranoside | 99.62 | 447.2233 | 10.17 | (M-H)- |  | √ | √ | √ | √ |  |  |  |  | √ |  |  |  |  |  |  |  |  |
| Gingerol | 99.89 | 293.175 | 11.57 | (M-H)- | √ | √ | √ | √ | √ | √ | √ | √ | √ | √ | √ | √ | √ | √ | √ | √ | √ | √ |
| Ginsenoside C-K | 99.25 | 621.4354 | 17.06 | (M-H)- |  | √ | √ | √ | √ |  |  | √ | √ | √ | √ | √ |  | √ | √ |  |  | √ |
| Glycyrrhetinic aldehyde | 99.53 | 453.3368 | 14.32 | (M+H)- | √ | √ | √ | √ | √ | √ | √ | √ | √ | √ | √ | √ | √ | √ | √ | √ | √ | √ |
| Gypsogenate | 99.38 | 531.3326 | 12.12 | (M+COOH)- | √ | √ | √ | √ | √ | √ | √ | √ | √ | √ | √ | √ | √ | √ | √ | √ | √ | √ |
| Hederagenin | 99.97 | 471.3469 | 13.30 | (M-H)- | √ | √ | √ | √ | √ | √ | √ | √ | √ | √ | √ | √ | √ | √ | √ | √ | √ | √ |
| Heptaketide pyrone | 99.13 | 321.0605 | 8.362 | (M+COOH)- | √ | √ | √ |  |  | √ |  |  |  |  |  |  | √ | √ |  | √ |  | √ |
| Jasmonate | 99.75 | 209.1167 | 10.93 | (M-H)- | √ | √ | √ | √ | √ | √ | √ | √ | √ | √ | √ | √ | √ | √ |  | √ |  |  |
| Leukoefdin | 97.71 | 321.0606 | 8.07 | (M-H)- | √ |  |  |  |  |  |  |  |  |  |  |  |  |  |  |  |  | √ |
| Linamarin | 99.28 | 246.0975 | 8.09 | (M-H)- | √ | √ | √ | √ | √ | √ | √ |  |  |  |  |  | √ | √ | √ | √ | √ | √ |
| Macarpine | 97.17 | 436.1042 | 7.96 | (M+COOH)- |  | √ |  |  | √ |  | √ | √ | √ | √ | √ | √ | √ | √ |  | √ | √ | √ |
| Mermesin | 99.87 | 245.0813 | 8.43 | (M-H)- | √ | √ | √ | √ | √ | √ | √ | √ | √ | √ | √ |  | √ | √ | √ | √ | √ | √ |
| Methylsalicyl-GXG | 95.97 | 653.1913 | 7.55 | (M+COOH)- | √ | √ | √ | √ | √ | √ |  |  |  |  |  |  |  | √ | √ | √ |  |  |
| Naringenin chalcone | 99.86 | 271.0607 | 10.63 | (M-H)- | √ | √ | √ | √ | √ | √ | √ | √ | √ | √ | √ | √ | √ | √ | √ | √ | √ | √ |
| Neolinustatin | 97.12 | 468.1711 | 8.10 | (M+COOH)- |  |  | √ | √ | √ | √ |  |  | √ | √ | √ | √ |  |  |  |  |  |  |
| Nothofagin | 99.94 | 435.1284 | 9.21 | (M-H)- | √ | √ | √ | √ | √ | √ | √ | √ | √ | √ | √ |  | √ | √ | √ | √ | √ | √ |
| Olivetol | 99.81 | 225.113 | 9.34 | (M+COOH)- |  | √ | √ | √ | √ | √ |  | √ | √ | √ | √ | √ |  |  |  |  |  |  |
| Phloretin | 99.07 | 273.0754 | 8.91 | (M-H)- | √ | √ | √ | √ | √ |  |  |  |  |  |  |  | √ |  | √ | √ |  | √ |
| Pinostrobin | 99.78 | 315.0864 | 9.21 | (M+COOH)- | √ | √ | √ | √ | √ | √ |  |  |  |  |  |  | √ | √ | √ | √ | √ | √ |
| Procyanidin B2 | 99.92 | 577.1338 | 8.22 | (M-H)- | √ | √ | √ | √ | √ | √ | √ | √ | √ | √ | √ | √ | √ | √ | √ | √ | √ | √ |
| Quercetagetin 7-O-glucoside | 99.72 | 479.0823 | 7.87 | (M-H)- | √ |  |  |  |  |  |  |  |  |  |  |  |  |  |  |  |  |  |
| Quercetin | 86.58 | 301.0346 | 9.55 | (M-H)- | √ | √ | √ | √ | √ | √ | √ |  |  |  |  |  |  |  |  | √ |  |  |
| Rishitin | 99.91 | 221.1539 | 11.57 | (M-H)- | √ | √ | √ | √ | √ | √ | √ | √ | √ | √ | √ | √ | √ | √ | √ | √ | √ | √ |
| salicyl-6-hydroxy-2-cyclohexene-on-oyl | 98.58 | 261.0757 | 7.87 | (M-H)- | √ | √ | √ | √ | √ | √ | √ | √ | √ | √ | √ | √ | √ | √ | √ |  | √ | √ |
| Sasanquin | 95.95 | 503.1737 | 10.56 | (M+COOH)- |  | √ |  |  |  |  |  |  |  |  |  |  |  |  |  |  |  |  |
| Sissotrin | 99.09 | 445.1131 | 8.84 | (M-H)- |  |  | √ | √ | √ | √ | √ | √ | √ | √ | √ | √ |  |  |  |  |  |  |
| Tetraketide pyrone | 99.07 | 223.0975 | 8.16 | (M-H)- |  |  |  |  | √ | √ |  |  |  |  | √ |  |  |  |  |  |  |  |
| trans-Tuberonic acid glucoside | 97.97 | 387.1653 | 8.41 | (M-H)- |  |  | √ | √ | √ | √ |  |  | √ | √ | √ | √ |  |  |  |  |  |  |
| Tuberonate | 99.81 | 225.113 | 9.34 | (M-H)- |  | √ | √ | √ | √ | √ |  | √ | √ | √ | √ | √ |  |  |  |  |  |  |
| Vanillate | 98.92 | 167.0343 | 9.34 | (M-H)- |  |  | √ |  |  |  | √ |  | √ |  |  |  | √ | √ | √ | √ | √ | √ |
| Vestitone | 97.70 | 331.0824 | 8.4 | (M+COOH)- |  |  |  |  |  | √ |  |  |  |  |  |  |  |  |  |  |  |  |
| Vincristine | 98.79 | 823.3891 | 9.60 | (M-H)- | √ | √ | √ | √ | √ | √ |  |  |  |  |  | √ |  |  |  |  |  |  |

**Table S3C. Putatively identified compounds at 30 days after MeJA elicitation (50 µM vs 100 µM vs ctrl), at different time points (0-72 hours).**

| **Methyl Jasmonate concentration (µM)** |  |  |  |  | **50** | | | | | | **100** | | | | | | **ctrl** | | | | | |
| --- | --- | --- | --- | --- | --- | --- | --- | --- | --- | --- | --- | --- | --- | --- | --- | --- | --- | --- | --- | --- | --- | --- |
| **Timeline (Hours)** |  |  |  |  | **0** | **6** | **12** | **24** | **48** | **72** | **0** | **6** | **12** | **24** | **48** | **72** | **0** | **6** | **12** | **24** | **48** | **72** |
| **Compound** | **Score** | **m/z** | **RT** | **Ion Specie** |  |  |  |  |  |  |  |  |  |  |  |  |  |  |  |  |  |  |
| (-)-Epicatechin gallate | 99.87 | 441.0806 | 9.17 | (M-H)- | √ | √ | √ | √ | √ | √ | √ | √ | √ | √ | √ | √ | √ | √ | √ | √ | √ | √ |
| (-)-Epigallocatechin gallate | 98.72 | 457.0762 | 8.75 | (M-H)- | √ | √ | √ | √ | √ | √ | √ | √ | √ | √ | √ |  | √ | √ | √ | √ | √ | √ |
| (+)-Artemisinate | 99.94 | 279.1607 | 11.60 | (M+COOH)- |  | √ |  |  | √ | √ | √ | √ | √ | √ |  | √ | √ | √ | √ | √ |  |  |
| (+)-Secoisolariciresinol | 99.58 | 407.1175 | 10.10 | (M+COOH)- | √ | √ |  |  | √ | √ | √ | √ | √ |  |  | √ |  |  |  | √ |  | √ |
| (+)-Taxifolin | 99.19 | 303.0501 | 9.470 | (M-H)- | √ | √ | √ | √ | √ | √ | √ | √ | √ | √ | √ | √ | √ | √ | √ | √ | √ | √ |
| (2R,3S,4S)-Leucocyanidin | 99.94 | 455.3523 | 14.73 | (M-H)- | √ | √ | √ | √ | √ | √ | √ | √ | √ | √ | √ | √ | √ | √ | √ | √ | √ | √ |
| (2S,4As,6aS,6bR,10S,12aS)-10-hydroxy-2-(hydroxymethyl)-2,4a,6a,6b,9,9,12a-heptamethyl-3,4,5,6,6a,7,8,8a,10,11,12,14b-dodecahydro-1H-picen-13-one | 99.94 | 455.3523 | 14.73 | (M-H)- | √ | √ | √ | √ | √ | √ | √ | √ | √ | √ | √ | √ | √ | √ | √ | √ | √ | √ |
| (E)-6'-Hydroxyferulate | 98.24 | 209.0449 | 8.14 | (M-H)- | √ | √ | √ | √ | √ | √ | √ | √ | √ | √ | √ | √ | √ | √ | √ | √ | √ | √ |
| (S)-laudanosine | 99.94 | 356.1861 | 11.27 | (M-H)- |  |  |  | √ | √ | √ |  |  |  | √ | √ | √ |  |  |  |  |  |  |
| 1,2,6-Trigalloylglucose | 95.91 | 635.0881 | 8.64 | (M-H)- |  |  |  |  |  |  |  | √ |  |  |  |  |  |  |  |  |  |  |
| 1,6-Bis-O-galloyl-beta-D-glucose | 98.82 | 483.0771 | 8.22 | (M-H)- |  | √ |  |  | √ |  |  | √ |  |  |  |  |  |  |  |  |  |  |
| 16-alpha-Hydroxygypsogenate | 92.44 | 501.3211 | 11.30 | (M+COOH)- | √ | √ | √ | √ | √ | √ | √ | √ | √ | √ | √ | √ | √ | √ | √ | √ | √ | √ |
| 1-O-Vanilloyl-beta-D-glucose | 99.87 | 329.0868 | 8.14 | (M-H)- | √ | √ | √ | √ | √ | √ | √ | √ | √ | √ | √ | √ | √ | √ | √ | √ | √ | √ |
| 2-Coumarate | 99.77 | 163.0395 | 8.99 | (M-H)- | √ | √ |  |  |  | √ | √ |  | √ | √ | √ | √ |  | √ | √ |  | √ |  |
| 2'-Hydroxy 3,6,7,3',4'-pentamethylquercetagetin | 99.71 | 449.1075 | 9.25 | (M+COOH)- | √ | √ | √ | √ | √ | √ | √ | √ | √ | √ | √ | √ | √ | √ | √ | √ | √ | √ |
| 2-Hydroxy-2,3-dihydrogenistein-7-olate | 99.81 | 287.0555 | 9.92 | (M-H)- | √ | √ | √ | √ | √ | √ | √ | √ | √ | √ | √ | √ | √ | √ | √ | √ | √ | √ |
| 2-Methoxycarbonylphenyl b-D-glucopyranoside | 99.91 | 359.0973 | 8.17 | (M+COOH)- | √ | √ | √ | √ | √ | √ | √ | √ | √ | √ | √ | √ | √ | √ | √ | √ | √ | √ |
| 3',5'-Di-C-glucosylphloretin | 99.76 | 643.1876 | 7.92 | (M+COOH)- | √ | √ | √ | √ | √ | √ | √ | √ | √ | √ | √ | √ | √ | √ | √ | √ | √ | √ |
| 3-beta-Hydroxyglycyrrhetinate | 99.93 | 515.3365 | 12.57 | (M+COOH)- | √ | √ | √ | √ | √ | √ | √ | √ | √ | √ | √ | √ | √ | √ | √ | √ | √ | √ |
| 3-Hydroxy-5-methoxybiphenyl | 99.87 | 245.0813 | 8.43 | (M+COOH)- | √ | √ | √ | √ | √ | √ | √ | √ | √ | √ | √ | √ | √ | √ | √ | √ | √ | √ |
| 3-Hydroxy-beta-ionone | 99.38 | 253.1439 | 11.29 | (M+COOH)- | √ | √ | √ | √ | √ | √ | √ | √ | √ | √ | √ | √ | √ | √ | √ | √ | √ | √ |
| 3-Isobutanoyl-3',4-di(isovaleryl)sucrose | 97.90 | 579.2644 | 8.90 | (M+COOH)- |  |  |  | √ | √ | √ |  |  |  | √ | √ | √ |  |  |  |  |  |  |
| 3-Lauryl-3'-isobutanoyl-4-(isovaleryl)sucrose | 99.36 | 723.3783 | 11.96 | (M+COOH)- | √ | √ | √ | √ | √ | √ | √ | √ | √ | √ | √ | √ | √ | √ | √ | √ | √ | √ |
| 3''-O-(4-Hydroxycinnamoyl)astragalin | 99.93 | 593.1282 | 8.20 | (M-H)- | √ | √ | √ | √ | √ | √ | √ | √ | √ | √ | √ | √ | √ | √ | √ | √ | √ | √ |
| 4alpha-Carboxy-5alpha-cholesta-7,24-dien-3beta-ol | 99.90 | 473.327 | 11.77 | (M+COOH)- | √ | √ | √ | √ | √ | √ | √ | √ | √ | √ | √ | √ | √ | √ | √ | √ | √ | √ |
| 4alpha-Carboxy-ergosta-7,24(241)-dien-3beta-ol | 99.90 | 487.3419 | 11.77 | (M+COOH)- | √ | √ | √ | √ | √ | √ | √ | √ | √ | √ | √ | √ | √ | √ | √ | √ | √ | √ |
| 4alpha-Formyl-ergosta-7,24(241)-dien-3beta-ol | 99.97 | 471.3469 | 13.30 | (M+COOH)- | √ | √ | √ | √ | √ | √ | √ | √ | √ | √ | √ | √ | √ | √ | √ | √ | √ | √ |
| 4alpha-Hydroxymethyl-4beta-methyl-5alpha-cholesta-8,24-dien-3beta-ol | 99.35 | 473.3623 | 12.62 | (M+COOH)- | √ | √ | √ | √ | √ | √ | √ | √ | √ | √ | √ | √ | √ | √ | √ | √ | √ | √ |
| 5-O-Caffeoylshikimate | 98.87 | 335.0763 | 8.46 | (M-H)- |  |  |  |  |  |  | √ | √ | √ |  |  |  |  |  |  |  |  |  |
| 9,10,18-Trihydroxystearate | 93.48 | 331.2479 | 11.40 | (M-H)- |  |  |  |  | √ |  |  |  |  |  |  |  |  |  |  |  |  |  |
| 9'-Hoaba | 89.24 | 279.121 | 8.89 | (M-H)- |  |  |  |  |  |  |  | √ | √ | √ | √ | √ |  |  |  |  |  |  |
| Abscisic alcohol | 99.93 | 249.1488 | 13.85 | (M-H)- | √ | √ | √ | √ | √ | √ | √ | √ | √ | √ | √ | √ | √ | √ | √ | √ | √ | √ |
| Alizarin | 90.40 | 285.0401 | 9.35 | (M+COOH)- | √ | √ | √ | √ | √ | √ |  | √ | √ | √ | √ | √ | √ | √ |  | √ | √ |  |
| alpha-3',4'-Anhydrovinblastine radical | 96.20 | 791.3994 | 10.22 | (M-H)- |  |  |  | √ | √ |  |  |  | √ | √ | √ | √ |  |  |  |  |  |  |
| Arctigenin | 100.00 | 371.1512 | 15.24 | (M-H)- | √ | √ | √ | √ | √ | √ | √ | √ | √ | √ | √ | √ | √ | √ | √ | √ | √ | √ |
| Artemisinin | 97.21 | 281.1391 | 9.48 | (M-H)- |  |  |  |  |  |  |  |  |  | √ | √ |  |  |  |  |  |  |  |
| Astilbin (Taxifolin 3-O-rhamnoside) | 99.71 | 449.1075 | 9.27 | (M-H)- | √ | √ | √ | √ | √ | √ | √ | √ | √ | √ | √ | √ | √ | √ | √ | √ | √ | √ |
| Avenastenone | 99.94 | 455.3523 | 14.72 | (M+COOH)- | √ | √ | √ | √ | √ | √ | √ | √ | √ | √ | √ | √ | √ | √ | √ | √ | √ | √ |
| Benzoyl-beta-D-glucoside | 99.87 | 329.0868 | 8.14 | (M+COOH)- | √ | √ | √ | √ | √ | √ | √ | √ | √ | √ | √ | √ | √ | √ | √ | √ | √ | √ |
| Bergapten | 84.80 | 215.0341 | 7.87 | (M+COOH)- |  |  |  |  |  |  |  |  |  |  |  |  | √ |  |  | √ | √ |  |
| Biliverdine | 86.60 | 581.2372 | 9.09 | (M-H)- |  |  | √ | √ | √ | √ |  | √ | √ | √ | √ | √ |  |  |  |  |  |  |
| C19-Gibberellin skeleton | 80.29 | 361.1627 | 11.56 | (M+COOH)- |  |  |  |  | √ | √ |  |  |  | √ | √ |  | √ |  |  |  |  |  |
| Catechin | 99.75 | 289.0711 | 8.50 | (M-H)- | √ | √ | √ | √ | √ | √ | √ | √ | √ | √ | √ | √ | √ | √ | √ | √ | √ | √ |
| Chalconaringenin 4'-glucoside | 97.12 | 433.1125 | 9.62 | (M-H)- | √ | √ | √ | √ | √ | √ | √ | √ | √ | √ | √ |  | √ | √ | √ | √ | √ | √ |
| cis-Zeatin | 93.36 | 218.1026 | 7.65 | (M-H)- |  |  |  |  | √ | √ |  |  |  |  | √ | √ |  |  |  |  |  |  |
| cis-zeatin-7-N-glucoside | 93.43 | 380.1588 | 14.68 | (M-H)- | √ | √ | √ | √ | √ | √ |  | √ | √ | √ | √ | √ | √ | √ | √ | √ | √ | √ |
| Combretol | 97.12 | 433.1125 | 9.62 | (M+COOH)- | √ | √ | √ | √ | √ | √ | √ | √ | √ | √ | √ |  | √ | √ | √ | √ | √ | √ |
| Coproporphyrinogen III | 94.56 | 659.3132 | 14.72 | (M-H)- | √ | √ | √ | √ |  | √ |  | √ | √ | √ | √ | √ | √ | √ |  | √ | √ | √ |
| Cyanidin 3-O-beta-D-caffeoylglucoside | 99.18 | 609.1226 | 7.84 | (M-H)- | √ | √ | √ | √ | √ | √ | √ | √ | √ | √ | √ | √ | √ | √ | √ | √ | √ | √ |
| Daphnetin | 99.90 | 177.0188 | 9.59 | (M-H)- |  |  |  |  |  |  |  | √ |  |  |  |  |  | √ |  | √ |  | √ |
| Dhurrin | 97.10 | 310.09 | 9.39 | (M-H)- |  |  |  | √ |  | √ |  | √ | √ | √ | √ | √ |  |  |  |  |  |  |
| Dicrocin | 99.29 | 651.2668 | 9.44 | (M+COOH)- | √ |  |  |  |  | √ | √ |  | √ | √ | √ | √ | √ |  | √ | √ |  |  |
| Dihydroprecondylocarpine acetate | 94.12 | 441.202 | 15.39 | (M-H)- | √ | √ | √ | √ | √ | √ |  |  | √ | √ | √ | √ | √ | √ | √ | √ | √ | √ |
| Diphyllin | 99.10 | 425.0863 | 8.02 | (M+COOH)- | √ | √ | √ | √ | √ | √ | √ | √ | √ | √ | √ | √ | √ | √ | √ | √ | √ | √ |
| Eugenyl-GXG | 94.67 | 665.2251 | 8.58 | (M+COOH)- |  |  |  |  | √ |  |  |  |  |  | √ | √ |  |  |  |  |  |  |
| Gaultherin | 99.90 | 491.1391 | 8.02 | (M-H)- | √ | √ | √ | √ | √ | √ | √ | √ | √ | √ | √ | √ | √ | √ | √ | √ | √ | √ |
| Geranyl 6-O-beta-D-xylopyranosyl-beta-D-glucopyranoside | 99.62 | 447.2233 | 10.17 | (M-H)- |  |  |  |  |  |  |  |  | √ | √ |  |  |  |  |  |  |  |  |
| Gingerol | 99.89 | 293.175 | 11.57 | (M-H)- |  | √ |  |  | √ | √ | √ | √ | √ | √ | √ | √ | √ | √ | √ | √ | √ | √ |
| Ginsenoside C-K | 99.25 | 621.4354 | 17.06 | (M-H)- | √ | √ | √ | √ | √ | √ |  | √ | √ | √ |  |  | √ | √ | √ | √ |  | √ |
| Ginsenoside rb1 | 88.19 | 1153.601 | 11.24 | (M-H)- | √ | √ | √ | √ | √ | √ | √ | √ | √ | √ | √ | √ | √ | √ | √ | √ | √ | √ |
| Glycyrrhetinic aldehyde | 99.53 | 453.3368 | 14.32 | (M+H)- | √ | √ | √ | √ | √ | √ | √ | √ | √ | √ | √ | √ | √ | √ | √ | √ | √ | √ |
| Gypsogenate | 99.38 | 531.3326 | 12.12 | (M+COOH)- | √ | √ | √ | √ | √ | √ | √ | √ | √ | √ | √ | √ | √ | √ | √ | √ | √ | √ |
| Hederagenin | 99.97 | 471.3469 | 13.30 | (M-H)- | √ | √ | √ | √ | √ | √ | √ | √ | √ | √ | √ | √ | √ | √ | √ | √ | √ | √ |
| Heptaketide pyrone | 99.13 | 321.0605 | 8.36 | (M+COOH)- |  | √ |  |  |  |  |  | √ | √ |  |  |  |  |  |  |  |  |  |
| Jasmonate | 99.75 | 209.1167 | 10.93 | (M-H)- | √ | √ | √ | √ | √ | √ | √ | √ | √ | √ | √ | √ | √ |  |  |  | √ |  |
| Leukoefdin | 97.71 | 321.0606 | 8.07 | (M-H)- |  |  |  |  |  |  |  | √ |  |  |  |  |  | √ |  |  |  |  |
| Linamarin | 99.28 | 246.0975 | 8.09 | (M-H)- | √ | √ | √ | √ | √ | √ | √ | √ | √ | √ | √ | √ | √ | √ | √ | √ | √ | √ |
| Lotaustralin | 98.37 | 260.1137 | 8.75 | (M-H)- |  |  |  |  | √ | √ |  |  |  |  |  |  |  |  |  |  |  |  |
| Macarpine | 97.17 | 436.1042 | 7.96 | (M+COOH)- |  |  |  |  | √ |  | √ | √ | √ |  | √ | √ |  |  |  |  |  |  |
| Mermesin | 99.87 | 245.0813 | 8.43 | (M-H)- | √ | √ | √ | √ | √ | √ | √ | √ | √ | √ | √ | √ | √ | √ | √ | √ | √ | √ |
| Methylsalicyl-GXG | 95.97 | 653.1913 | 7.55 | (M+COOH)- | √ |  | √ | √ | √ | √ |  | √ | √ | √ | √ | √ | √ | √ | √ | √ | √ | √ |
| Naringenin chalcone | 99.86 | 271.0607 | 10.63 | (M-H)- | √ |  | √ |  | √ | √ | √ | √ | √ | √ | √ | √ | √ | √ | √ | √ | √ | √ |
| Neolinustatin | 97.12 | 468.1711 | 8.10 | (M+COOH)- |  |  |  | √ | √ | √ |  |  |  | √ | √ | √ |  |  |  |  |  |  |
| Nothofagin | 99.94 | 435.1284 | 9.21 | (M-H)- | √ | √ | √ | √ | √ | √ | √ | √ | √ | √ | √ | √ | √ | √ | √ | √ | √ | √ |
| Olivetol | 99.81 | 225.113 | 9.34 | (M+COOH)- |  | √ | √ | √ | √ | √ |  | √ | √ | √ | √ | √ |  |  |  |  |  |  |
| Phloretin | 99.07 | 273.0754 | 8.91 | (M-H)- | √ | √ | √ | √ | √ | √ | √ | √ |  |  | √ | √ | √ | √ | √ | √ | √ | √ |
| Pinostrobin | 99.78 | 315.0864 | 9.21 | (M+COOH)- | √ | √ | √ | √ | √ | √ | √ | √ | √ | √ | √ | √ | √ | √ | √ | √ | √ | √ |
| Procyanidin B2 | 99.92 | 577.1338 | 8.22 | (M-H)- | √ | √ | √ | √ | √ | √ | √ | √ | √ | √ | √ | √ | √ | √ | √ | √ | √ | √ |
| Quercetin | 86.58 | 301.0346 | 9.55 | (M-H)- |  | √ |  |  |  | √ |  |  |  |  |  |  |  |  |  |  | √ |  |
| Rishitin | 99.91 | 221.1539 | 11.57 | (M-H)- | √ | √ | √ | √ | √ | √ | √ | √ | √ | √ | √ | √ | √ | √ | √ | √ | √ | √ |
| salicyl-6-hydroxy-2-cyclohexene-on-oyl | 98.58 | 261.0757 | 7.87 | (M-H)- | √ | √ | √ | √ | √ | √ | √ | √ | √ | √ | √ | √ | √ | √ | √ | √ | √ | √ |
| Sissotrin | 99.09 | 445.1131 | 8.84 | (M-H)- |  |  |  | √ | √ |  |  | √ |  | √ | √ | √ |  |  |  |  |  |  |
| Tetraketide pyrone | 99.07 | 223.0975 | 8.16 | (M-H)- |  |  |  |  |  | √ |  |  |  | √ | √ | √ |  |  |  |  |  |  |
| trans-Tuberonic acid glucoside | 97.97 | 387.1653 | 8.41 | (M-H)- |  |  | √ |  | √ | √ |  |  | √ | √ | √ | √ |  |  |  |  |  |  |
| Tuberonate | 99.81 | 225.113 | 9.34 | (M-H)- |  | √ | √ | √ | √ | √ |  | √ | √ | √ | √ | √ |  |  |  |  |  |  |
| Vanillate | 98.92 | 167.0343 | 9.34 | (M-H)- | √ |  |  |  |  |  |  | √ |  |  |  |  |  | √ | √ | √ | √ | √ |
| Vernolic acid | 99.05 | 341.2331 | 11.01 | (M+COOH)- |  |  |  |  |  |  | √ |  |  |  |  |  |  |  |  |  |  |  |
| Vestitone | 97.70 | 331.0824 | 8.40 | (M+COOH)- |  |  |  | √ |  |  |  |  |  | √ | √ |  |  |  |  |  |  |  |

**Table S3D. Putatively identified compounds in Feijoa floral buds.**

| **Compound** | **Score** | **m/z** | **RT** | **Ion Specie** | **Bud** |
| --- | --- | --- | --- | --- | --- |
| (-)-Epicatechin gallate | 79.66 | 441.0824 | 9.31 | (M-H)- | √ |
| (-)-Epigallocatechin gallate | 66.64 | 457.0781 | 8.65 | (M-H)- | √ |
| (+)-Artemisinate | 82.32 | 279.1607 | 11.60 | (M+COOH)- | √ |
| (+)-Secoisolariciresinol | 92.94 | 407.1175 | 10.10 | (M+COOH)- | √ |
| (+)-Taxifolin | 96.72 | 349.0569 | 9.32 | (M-H)- | √ |
| 1,2,6-Trigalloylglucose | 80.65 | 681.0932 | 8.72 | (M-H)- | √ |
| 1,6-Bis-O-galloyl-beta-D-glucose | 91.41 | 483.0782 | 8.35 | (M-H)- | √ |
| 2-Coumarate | 87.33 | 163.0398 | 8.22 | (M-H)- | √ |
| 2-Hydroxy-2,3-dihydrogenistein-7-olate | 85.11 | 287.0563 | 9.96 | (M-H)- | √ |
| 3,5-Didehydroshikimate | 91.86 | 169.014 | 4.59 | (M-H)- | √ |
| 3-Hydroxy-beta-ionone | 81.27 | 253.1445 | 11.54 | (M+COOH)- | √ |
| 4alpha-Carboxy-5alpha-cholesta-7,24-dien-3beta-ol | 86.87 | 473.327 | 11.77 | (M+COOH)- | √ |
| 4-Coumaroylshikimate | 85.89 | 319.0831 | 8.77 | (M-H)- | √ |
| 6-O-Galloyl-beta-D-glucose | 99.35 | 331.0674 | 4.46 | (M-H)- | √ |
| Alizarin | 85.23 | 285.0406 | 9.55 | (M+COOH)- | √ |
| Arctigenin | 100 | 371.1539 | 15.40 | (M-H)- | √ |
| Benzoyl-beta-D-glucoside | 82.52 | 343.1031 | 8.22 | (M+COOH)- | √ |
| cis-Zeatin | 88.45 | 218.1035 | 7.79 | (M-H)- | √ |
| Combretol | 63.52 | 433.113 | 9.20 | (M+COOH)- | √ |
| Gentisate 5-O-beta-D-xylopyranoside | 99.35 | 331.0674 | 4.46 | (M+COOH)- | √ |
| Gingerol | 95.3 | 293.1764 | 11.81 | (M-H)- | √ |
| Mermesin | 81.43 | 245.0819 | 8.55 | (M-H)- | √ |
| Phloretin | 96.29 | 319.0831 | 8.77 | (M-H)- | √ |
| Quercetin | 94.1 | 301.0352 | 9.91 | (M-H)- | √ |
| Rishitin | 82.65 | 221.1546 | 11.81 | (M-H)- | √ |
